# Psoriasis as a spatial immune ecosystem: insights from single-cell and spatial transcriptomics

**DOI:** 10.3389/fimmu.2026.1915876

**Published:** 2026-08-06

**Authors:** Wenkang Cheng, Ting Luo, Xiaoyu Zhou, Haixia Jing

**Affiliations:** Department of Dermatology, Taihe Hospital, Hubei University of Medicine, Shiyan, Hubei, China

**Keywords:** cell-cell communication, fibroblasts, immune heterogeneity, precision medicine, psoriasis, single-cell RNA sequencing, spatial transcriptomics, tissue microenvironment

## Abstract

Psoriasis is a chronic systemic inflammatory skin disease marked by substantial heterogeneity in both clinical presentation and immunopathology. Bulk RNA sequencing has been instrumental in establishing the IL-23/IL-17 axis as a central pathogenic framework, but tissue-averaged signals may obscure the cell-state heterogeneity and spatial microenvironments that distinguish clinical subtypes at single-cell resolution. Recent advances in single-cell RNA sequencing (scRNA-seq) and spatial transcriptomics (ST) have therefore prompted a conceptual shift from a model centered on individual cytokines toward a multidimensional, dynamic tissue ecosystem. This review synthesizes the latest spatiotemporal multi-omics evidence across key subtypes, including plaque, guttate, generalized pustular, palmoplantar pustulosis, erythrodermic psoriasis, and psoriatic arthritis. Emerging findings indicate that the distinct pathogeneses of these subtypes may arise from specific cell-state transitions and spatially restricted pathological niches shaped by epidermal inflammation, dermal immune activity, stromal remodeling, and neurovascular crosstalk. Keratinocytes and fibroblasts have moved beyond their traditionally passive roles as target cells; they are now recognized as active contributors that may help shape, anchor, and amplify the local inflammatory microenvironment through ligand–receptor communication axes. Longitudinal omics studies of targeted biologics further point to a temporal cascade model in which tissue microenvironment remodeling may precede the secondary deactivation of adaptive immunity. Even after clinical lesion resolution, a residual inflammatory milieu mediated by tissue-resident memory cells and memory-like fibroblasts may contribute to disease memory and local recurrence. We propose that future precision medicine strategies will need to look beyond peripheral clinical scores such as PASI and work toward a spatiotemporal multi-omics translational framework centered on histological deep remission and comprehensive pathological niche remodeling. Such a framework could provide systems-level biological guidance with the ultimate aim of advancing toward more durable disease control and deeper molecular remission in psoriasis.

## 1 Introduction

Psoriasis is a chronic, relapsing, systemic inflammatory disease associated with immune dysregulation (1). It is typically characterized by aberrant keratinocyte proliferation and impaired differentiation, leading to well-demarcated, erythematous, scaly plaques commonly affecting the scalp, elbows, and knees, often accompanied by pruritus and a burning sensation (2, 3). Beyond cutaneous involvement, psoriasis is closely associated with a range of systemic conditions, including psoriatic arthritis, cardiovascular and cerebrovascular diseases, diabetes mellitus, metabolic syndrome, obesity, osteoporosis, inflammatory bowel disease, uveitis, and hepatic or renal dysfunction; it is now increasingly recognized as a systemic inflammatory disorder (4). Transcriptomic technologies have significantly advanced research into the pathogenesis of psoriasis. Conventional bulk RNA sequencing, by averaging signals across tissue samples, has helped establish the classic pathogenic model centered on the IL-23/IL-17 axis, TNF signaling, and interferon-related inflammatory responses (5, 6). However, psoriatic lesions are inherently complex microenvironments composed of keratinocytes, T cells, dendritic cells, macrophages, neutrophils, endothelial cells, fibroblasts, and other cell types (7, 8). Bulk transcriptomic approaches are often unable to resolve the specific transcriptional signatures of distinct cell populations and may fail to identify low-abundance yet functionally critical cell subsets, thereby limiting a more refined understanding of disease heterogeneity (9).

**Figure 1:**
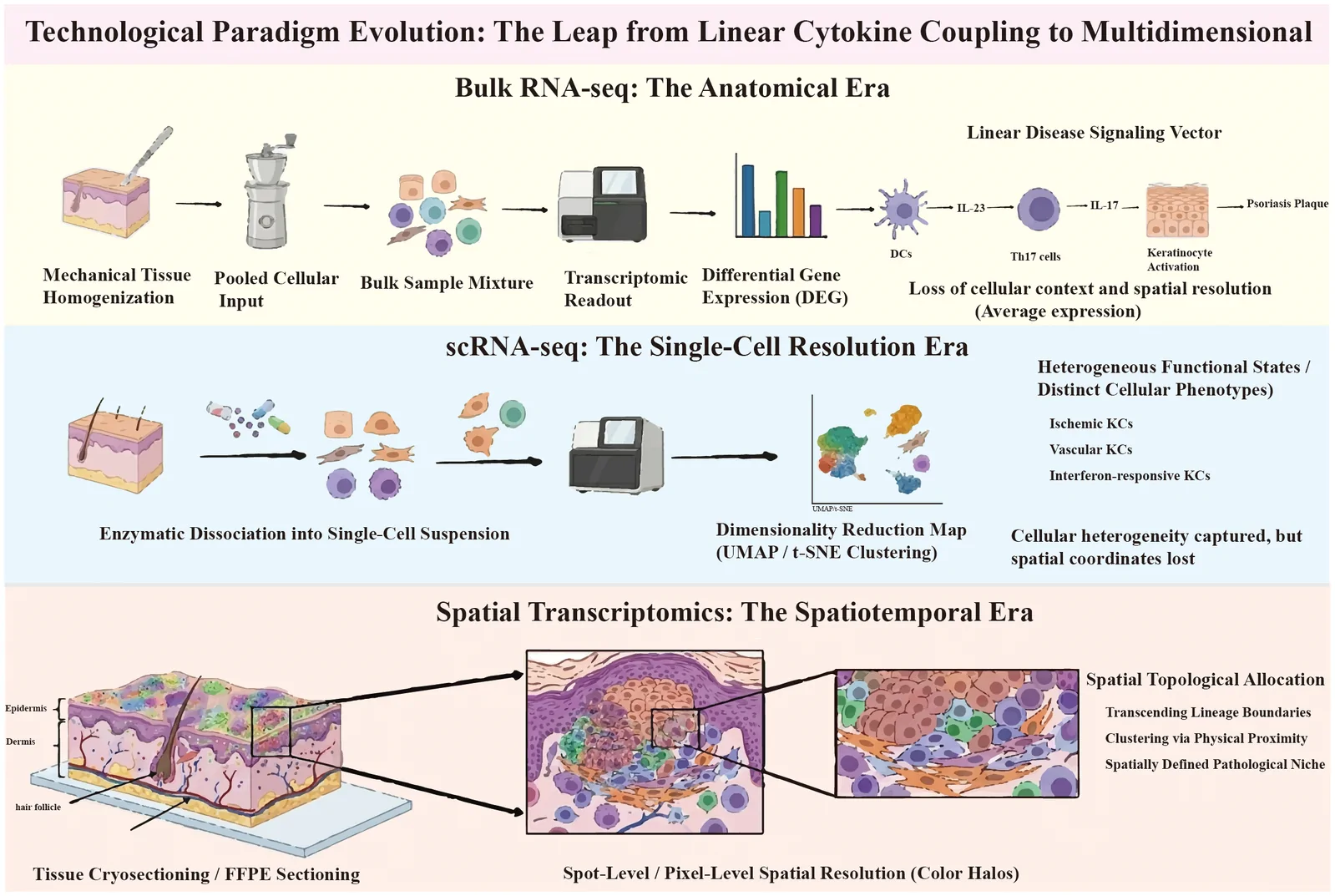
Evolving technological paradigms in psoriasis research: from bulk transcriptomics to single-cell and spatial ecosystem dissection. Bulk RNA sequencing (Bulk RNA-seq) helped establish the canonical IL-23/IL-17 pathogenic model, but is often limited by tissue-averaged signals and may not adequately resolve cellular heterogeneity or spatial architecture. Single-cell RNA sequencing (scRNA-seq) enables precise identification of cell states and pathogenic subpopulations, although tissue dissociation results in the loss of spatial information. Spatial transcriptomics further integrates molecular information with anatomical coordinates, allowing the reconstruction of *in situ* pathological niches, cell-cell communication networks, and microenvironmental interactions, and is driving a transition in psoriasis research from a linear cytokine model toward a multidimensional immune-stromal ecosystem model.

The emergence of scRNA-seq and ST has opened new avenues for dissecting the intricate microenvironment of psoriasis (8). scRNA-seq enables the characterization of cell-state transitions and subpopulation heterogeneity at single-cell resolution, whereas ST preserves *in situ* spatial information, allowing the mapping of cell-type distribution and interaction patterns within the epidermis, dermis, perivascular regions, and skin appendages (10–13). Increasing evidence suggests that the initiation, maintenance, and recurrence of psoriasis are not attributable to a single immune cell type alone, but are shaped by complex cellular communication networks mediated by cytokines, chemokines, and ligand-receptor interactions (7). Integrating scRNA-seq and ST in subtype-specific investigations holds considerable promise for elucidating the immunological heterogeneity and key cellular niches of psoriasis. Such integrative approaches may also provide an important theoretical foundation for precise disease classification, prediction of therapeutic response, and the discovery of novel therapeutic targets, thereby helping to advance psoriasis research into the era of precision medicine (14, 15) (**Figure 1**).

## 2 Literature search and conceptual framework

To capture the rapidly evolving spatiotemporal immune landscape of psoriasis in a comprehensive yet focused manner, we conducted a targeted literature search across PubMed, Embase, and Web of Science from database inception to June 2026. The search strategy combined Medical Subject Headings (MeSH) and free-text keywords, including “psoriasis”, “single-cell RNA sequencing”, “spatial transcriptomics”, “cell-cell communication”, and “tissue microenvironment”. Although this article is a narrative synthesis rather than a formal systematic review or meta-analysis, priority was given to peer-reviewed, high-resolution multi-omics studies that delineate cellular niches and tissue architecture. This structured approach is intended to ensure that the reviewed evidence is both timely and methodologically sound, providing a reliable foundation for the multidimensional landscape analysis that follows.

### 2.1 Technical considerations and limitations of single-cell and spatial transcriptomics

Single-cell RNA sequencing and spatial transcriptomics provide complementary approaches for characterizing cellular heterogeneity and tissue organization in psoriasis. However, findings generated by these platforms are influenced by several technical and computational factors and should therefore be interpreted within the context of their methodological limitations.

A major challenge in scRNA-seq studies is the presence of batch effects arising from differences in patient characteristics, anatomical sampling sites, disease stages, treatment exposure, tissue-processing protocols, sequencing platforms, and experimental batches (16). These technical sources of variation may obscure biologically meaningful differences or create apparent cell states that are not consistently reproduced across cohorts. Although computational integration methods can reduce unwanted variation, excessive batch correction may also remove genuine subtype-or disease-specific signals. Consequently, integrated analyses should retain biological replicates and carefully evaluate whether clinically relevant differences are preserved after correction.

Transcript dropout and limited RNA-capture efficiency represent additional challenges. Single-cell expression matrices are inherently sparse, and low-abundance transcripts may remain undetected even when they are biologically important (16). This issue is particularly relevant to psoriasis because pathogenic cytokines such as IL17A, IL23A, and several chemokines may be expressed transiently or by relatively small cellular populations. The absence of a transcript in an individual cell therefore does not necessarily indicate the absence of the corresponding biological activity. Pseudobulk analyses, protein-level validation, and integration across independent datasets may help reduce the risk of overinterpreting sparse single-cell signals.

Tissue dissociation can also introduce substantial bias. Enzymatic and mechanical processing may preferentially recover readily dissociable cells while underrepresenting fragile, strongly adherent, or anatomically restricted populations. Dissociation may additionally induce stress-response genes and alter the transcriptional states of keratinocytes, fibroblasts, endothelial cells, and immune cells. As a result, the cellular composition observed by scRNA-seq may not fully reflect the original tissue architecture. Single-nucleus RNA sequencing can reduce some dissociation-associated effects, although it provides more limited information on cytoplasmic transcripts and may introduce its own cell-type-specific biases (17, 18).

Spatial transcriptomic technologies preserve anatomical context but differ substantially in spatial resolution, transcript coverage, and detection sensitivity. Spot-based platforms may capture transcripts from multiple neighboring cells, particularly at the dermal-epidermal junction and within densely populated inflammatory niches. Cell-type assignments in these regions frequently depend on computational deconvolution rather than direct single-cell measurement. Higher-resolution and imaging-based platforms can improve cellular localization, but some approaches remain restricted by predefined gene panels, lower transcript coverage, limited tissue area, or high analytical cost. Moreover, spatial colocalization does not by itself demonstrate direct cellular interaction or functional signaling. Computational variability is another important source of uncertainty. Cell filtering, normalization, dimensionality reduction, clustering resolution, marker selection, cell-type annotation, trajectory inference, spatial deconvolution, and ligand-receptor analysis can produce different results when alternative pipelines or parameter settings are used. The nomenclature assigned to dendritic-cell and fibroblast subsets is therefore not always directly comparable across studies. Similarly, predicted developmental trajectories and intercellular communication networks should be considered hypothesis-generating models rather than definitive evidence of lineage relationships or molecular interactions (19, 20).

Reproducibility is further limited by small sample sizes, incomplete clinical metadata, unequal representation of psoriasis subtypes, and a shortage of independent validation cohorts. Future studies should adopt standardized tissue-processing and analytical protocols, include sufficient biological replicates, report detailed clinical and technical metadata, and make raw data, analytical code, and parameter settings publicly available. Orthogonal validation using multiplex imaging, proteomics, flow cytometry, *in situ* hybridization, co-culture assays, and functional perturbation models will be essential for distinguishing reproducible biological mechanisms from platform-specific or computationally derived observations. A comparative overview of the major single-cell and spatial transcriptomic platforms, including their principles, resolution, advantages, limitations, and suitability for psoriasis research, is provided in **Table 1**.

**Table 1:** Comparative characteristics of major single-cell and spatial transcriptomic platforms and their potential applications in psoriasis research.

Platform or approach	Basic principle	Cellular or spatial resolution	Major advantages	Principal limitations	Potential suitability for psoriasis research
10x Genomics Chromium 3′/5′ scRNA-seq (21)	Individual cells are partitioned into barcoded droplets, allowing high-throughput capture and sequencing of 3′ or 5′ transcript ends. The 5′ workflow can additionally support paired T-cell or B-cell receptor profiling.	Single-cell resolution; no spatial coordinates	High cellular throughput; suitable for identifying rare cell populations and comparing cellular composition across patients; mature analytical ecosystem; 5′ assays enable immune-receptor clonotype analysis	Requires viable cell suspensions; tissue dissociation may alter transcriptional states and underrepresent fragile or strongly adherent cells; relatively sparse transcript detection; limited isoform information	Well suited for constructing large-scale immune and stromal cell atlases, identifying pathogenic Tc17, TRM, dendritic-cell, myeloid, keratinocyte, and fibroblast states, and examining T-cell clonotype expansion across skin and joint tissues
Smart-seq3 (22)	Plate-based single-cell RNA sequencing combining full-length transcript coverage with unique molecular identifiers for transcript counting	Single-cell resolution; no spatial coordinates	High transcript-detection sensitivity; improved detection of low-abundance genes; enables analysis of transcript isoforms, allelic expression, and sequence variants	Lower cellular throughput than droplet-based methods; higher per-cell cost and sequencing demand; requires cell sorting and viable single-cells	Particularly useful for deeply profiling selected rare populations, such as pathogenic T-cell, dendritic-cell, or fibroblast subsets, and for examining low-abundance cytokines, transcription factors, and alternative transcript isoforms
Single-nucleus RNA sequencing (snRNA-seq) (18, 23)	RNA is isolated and sequenced from individual nuclei rather than intact cells, commonly using droplet- or combinatorial-indexing workflows	Single-nucleus resolution; no spatial coordinates	Compatible with frozen or difficult-to-dissociate tissues; reduces some enzymatic dissociation-associated stress responses; may improve recovery of strongly adherent stromal or structural populations	Lower abundance of cytoplasmic transcripts; enrichment of intronic reads; nuclear and cell-type-specific recovery biases; reduced detection of some activation-associated transcripts	Suitable for archived frozen skin, highly inflamed or fibrotic lesions, and studies focused on keratinocyte, fibroblast, endothelial, or other cell populations that may be poorly recovered by conventional tissue dissociation
10x Genomics Visium v1/v2 or CytAssist (19)	Tissue-derived transcripts are captured on spatially barcoded spots and sequenced, enabling whole-transcriptome expression profiles to be mapped onto histological images	Spot-level resolution; approximately 55-μm-diameter capture spots, commonly containing transcripts from multiple neighboring cells	Unbiased or broad transcriptome coverage; preserves tissue architecture; compatible with histological examination; suitable for comparing lesional, non-lesional, border, and resolving regions	Not true single-cell resolution; individual spots may contain multiple cell types; requires computational deconvolution; limited sensitivity for rare cell populations and low-abundance transcripts	Appropriate for identifying epidermal, superficial dermal, deep dermal, vascular, and lesion-border transcriptional domains and for mapping broad inflammatory niches across psoriatic plaques
10x Genomics Visium HD (20)	Whole-transcriptome expression is captured on a continuous array of spatially barcoded 2 × 2-μm squares and subsequently analyzed using binning or image-based cell segmentation	2-μm barcoded squares; commonly aggregated into larger bins or segmented to approximate single-cell-scale profiles	Whole-transcriptome spatial profiling at substantially higher resolution than conventional Visium; compatible with matched histological images; enables detailed mapping of tissue boundaries and small pathological niches	Cellular assignment depends partly on image segmentation and computational aggregation; adjacent-cell transcripts may still be difficult to separate; large datasets and substantial computational resources are required	Highly suitable for resolving the dermal-epidermal junction, inflammatory expansion fronts, perivascular immune aggregates, fibroblast-rich stromal niches, and residual inflammatory memory in clinically resolved lesions
GeoMx Digital Spatial Profiler (24)	RNA probes or antibodies are linked to photocleavable oligonucleotide barcodes. Selected regions of interest or morphological compartments are illuminated, and released barcodes are quantified	Region-of-interest or segmented-compartment resolution rather than unbiased single-cell resolution	Compatible with FFPE and fresh-frozen tissue; permits simultaneous spatial RNA or protein profiling; allows morphology-guided selection of epidermal, dermal, vascular, or immune compartments; suitable for retrospective clinical cohorts	Results depend on predefined regions of interest; limited ability to discover unexpected spatial structures outside selected regions; cellular resolution depends on segmentation strategy; potential selection bias	Particularly useful for analyzing archived diagnostic biopsies, comparing epidermal and dermal compartments, validating candidate biomarkers in clinically annotated cohorts, and studying treatment-associated changes in selected pathological regions
10x Genomics Xenium In Situ (20, 25)	Targeted RNA molecules are detected directly within intact tissue using cyclic probe hybridization, amplification, imaging, and spatial decoding	Single-cell and subcellular resolution	High spatial precision; direct localization of individual transcripts; preserves cellular morphology; enables analysis of rare cells and close cellular neighborhoods; panels can include hundreds to several thousand genes	Primarily targeted rather than fully unbiased; results depend on panel design; cell segmentation may affect assignment of transcripts; specialized instrumentation and substantial cost	Suitable for validating the localization of TRM cells, LAMP3^+^ dendritic cells, inflammatory fibroblasts, and keratinocyte states and for examining predicted ligand-receptor pairs within epidermal, stromal, and perivascular niches
CosMx Spatial Molecular Imager (26)	Repeated high-plex *in situ* hybridization and imaging are used to detect RNA, with some assays also supporting protein measurement in the same tissue section	Single-cell and subcellular resolution	Very high RNA plex, including whole-transcriptome-scale assays; compatible with FFPE and fresh-frozen tissue; simultaneous spatial phenotyping of large numbers of cells; supports spatial RNA-protein analysis	High cost and analytical complexity; lengthy imaging workflows; cell segmentation and transcript assignment remain important sources of variability; large data-storage and computational requirements	Suitable for comprehensive spatial mapping of heterogeneous psoriatic lesions, identifying rare immune-stromal neighborhoods, and validating cell states and signaling programs in archival or prospectively collected biopsies
MERFISH and seqFISH^+^ (27)	Individual RNA molecules are assigned combinatorial barcodes and detected through sequential rounds of fluorescence *in situ* hybridization and imaging	Single-molecule, single-cell, and subcellular resolution	Direct visualization and counting of RNA molecules; high spatial precision; preserves tissue architecture; enables analysis of subcellular RNA localization and neighboring-cell relationships	Complex probe design and imaging workflows; specialized instrumentation and computational pipelines; optical crowding and tissue autofluorescence may affect performance; targeted gene selection is usually required	Valuable for high-resolution validation of low-abundance cytokines, chemokines, cell-state markers, and ligand-receptor pairs and for studying transcript localization within keratinocytes and closely apposed immune cells

This table summarizes the basic principles, cellular or spatial resolution, major advantages, principal limitations, and potential applications of commonly used single-cell and spatial transcriptomic approaches in psoriasis research. These platforms differ in transcriptome coverage, cellular throughput, tissue compatibility, spatial precision, and suitability for discovery or validation studies. Platform specifications, including gene-plex capacity and effective spatial resolution, may vary according to assay version, panel design, tissue quality, image segmentation, and analytical workflow. Abbreviations: scRNA-seq, single-cell RNA sequencing; snRNA-seq, single-nucleus RNA sequencing; TRM, tissue-resident memory T cell; Tc17, interleukin-17-producing CD8^+^ T cell; FFPE, formalin-fixed paraffin-embedded; ROI, region of interest; MERFISH, multiplexed error-robust fluorescence *in situ* hybridization; seqFISH+, sequential fluorescence *in situ* hybridization plus.

## 3 Clinical subtypes and immunopathological heterogeneity of psoriasis

Psoriasis is not a single disease but a heterogeneous disease spectrum that shares a common genetic predisposition yet exhibits diverse clinical phenotypes and immune drivers. Recent applications of scRNA-seq and ST indicate that different subtypes may display marked differences in immune cell composition, inflammatory amplification circuits, and tissue microenvironment remodeling (7, 14, 28). In broad terms, plaque psoriasis is generally characterized by a chronic adaptive immune response linked to the IL-23/Th17 axis; guttate psoriasis often represents an infection-triggered disease initiation stage; generalized pustular psoriasis is largely considered an autoinflammatory condition associated with IL-36 pathway dysregulation; palmoplantar pustulosis appears to reflect localized tissue niche remodeling; erythrodermic psoriasis typically manifests as a systemic inflammatory decompensation state; and psoriatic arthritis further illustrates the systemic extension of inflammation from the skin to distal organs (5, 29–33). Subtype-specific cellular compositions, molecular pathways, spatial features, clinical implications, and remaining knowledge gaps are summarized in **Table 2**.

**Table 2:** Comparative immune-cell composition, molecular pathways, spatial features, clinical implications, and unresolved questions across the major clinical phenotypes of psoriatic disease.

Psoriasis subtype	Dominant immune populations	Key molecular pathways	Major spatial features	Clinical implications	Remaining knowledge gaps
Plaque psoriasis (PV) (5, 6, 14, 15, 28, 34–41)	Tc17, TRM cells, inflammatory fibroblasts, LAMP3^+^ DCs	IL-23/IL-17 axis, TNF, IL-36 amplification	Stable inflammatory niche with coordinated immune stromal interactions	Explains chronic inflammation and disease recurrence	Relative contributions of stromal versus immune compartments remain unclear; lack of longitudinal validation
Guttate psoriasis (GP) (31, 42)	Activated T cells, early dendritic cells, keratinocytes	IL-23/IL-17 signaling, type I interferon	Acute inflammatory lesions with evolving immune memory	Model for early psoriasis initiation	Whether GP represents a distinct entity or an early stage of PV remains unresolved
Generalized pustular psoriasis (GPP) (29, 43–49)	Neutrophils, macrophages, keratinocytes	IL-36 pathway, CXCL1/CXCL8, IL-17	Diffuse neutrophilic inflammation	Supports IL-36-targeted therapy	Mechanisms underlying disease heterogeneity beyond IL36RN mutations remain unclear
Palmoplantar pustulosis (PPP) (6, 30, 34–37, 50)	Tc17 cells, neutrophils, keratinocytes	IL-36, IL-17, TNF	Palmoplantar immune niche	Potentially distinct molecular subtype	Disease classification and subtype-specific mechanisms remain controversial
Erythrodermic psoriasis (EP) (32, 51)	Activated T cells, myeloid cells, keratinocytes	TNF, IL-17, IFN-related pathways	Systemic inflammatory remodeling	Reflects severe systemic immune dysregulation	Limited by small cohorts and lack of longitudinal multi-omics data
Psoriatic arthritis (PsA) (35, 50)	Tc17 cells, fibroblasts, macrophages, TRM cells	IL-17, TNF, JAK/STAT	Shared skin-joint immune ecosystem	Explains skin-joint immune crosstalk	Cellular migration routes and causal mechanisms linking skin and joint remain poorly defined

This table summarizes the predominant immune and stromal populations, inflammatory pathways, spatial features, clinical implications, and knowledge gaps reported for plaque psoriasis, guttate psoriasis, generalized pustular psoriasis, palmoplantar pustulosis, erythrodermic psoriasis, and psoriatic arthritis. Because evidence is uneven across phenotypes, the entries represent comparative patterns rather than exclusive or universally validated mechanisms.

### 3.1 Plaque psoriasis

Plaque psoriasis (psoriasis vulgaris, PV) is the most common subtype, and its core pathogenesis is thought to involve a dendritic-cell associated IL-23/IL-17 inflammatory network. External triggers such as infections, mechanical stress, and certain drugs are believed to first activate plasmacytoid dendritic cells (pDCs), inducing IFN-α release; subsequently, conventional dendritic cells secrete IL-23 and TNF-α, which are thought to promote the expansion of Th17 and Tc17 cells (35, 36). Activated Th17 cells produce IL-17A, IL-17F, and IL-22, which may promote aberrant keratinocyte proliferation and the release of additional inflammatory mediators, thereby contributing to a self-perpetuating chronic inflammatory loop (34). Keratinocytes are no longer viewed merely as passive targets of inflammation, but are increasingly recognized as important amplifiers that help sustain the disease. Upon stimulation with IL-17A and TNF-α, keratinocytes are thought to upregulate a range of pro-inflammatory molecules, including CXCL1, CXCL8, CCL20, S100A7, S100A8/S100A9, and IL36G, which are predicted to further recruit Th17 cells, neutrophils, and dendritic cells, potentially reinforcing a self-sustaining positive-feedback loop (39). In particular, the aberrant activation of IL-36γ is thought to potentiate IL-17 and TNF-mediated inflammatory responses, forming a multi-tiered amplification network IL-23/Th17/IL-17/keratinocyte/IL-36 that likely contributes to the persistence of chronic lesions (14, 49). Single-cell and spatial omics studies further indicate that PV is not attributable to a single inflammatory axis alone; rather, it appears to be maintained by a stable immune niche composed of keratinocytes, inflammatory fibroblasts, tissue-resident memory T cells, and LAMP3^+^ dendritic cells (14, 15, 28, 40, 52).

Studies consistently identify inflammatory fibroblasts, tissue-resident memory T (TRM) cells, and LAMP3^+^ dendritic cells within chronic plaque lesions, but their relative contributions may vary across disease stages. Reports emphasizing stromal remodeling versus persistent TRM programs differ in disease duration, biopsy site, treatment exposure, sequencing platform, and annotation strategy. Matched longitudinal sampling is needed to determine whether these components occupy distinct or sequential positions within the maintenance hierarchy.

### 3.2 Guttate psoriasis

Guttate psoriasis (GP) is a classic acute-onset form of psoriasis, typically developing 1–3 weeks after an upper respiratory streptococcal infection, and is often regarded as an ideal model for studying the initial stages of psoriasis pathogenesis (31, 42). Pathogen associated molecular patterns derived from Streptococcus can be recognized by keratinocytes, dendritic cells, and macrophages, leading to the release of IL-1β, IL-6, TNF-α, and IL-23. Concurrently, pDCs are thought to rapidly infiltrate the dermis and secrete IFN-α, thereby triggering the Th17 inflammatory axis (34). The classical molecular mimicry theory proposes that structural homology between streptococcal M protein and keratin may allow activated T cells to cross-react with self-antigens during pathogen clearance, breaking immune tolerance. Compared with PV, GP is thought to better reflect the early transition from a healthy state to an inflammatory state (42). Single-cell studies have revealed that early lesions are dominated by activated dendritic cells, neutrophils, and effector T cells with high IL17A expression, whereas TRM cells are relatively scarce (8, 39). As the disease progresses, some patients gradually establish a stable TRM reservoir and eventually convert to chronic PV (52, 53). GP may therefore be better understood as a critical transitional phase in the natural history of psoriasis, bridging acute inflammation and the establishment of chronic immunological memory.

Guttate psoriasis remains a valuable model for investigating early psoriatic inflammation, but whether it is a distinct entity or a transitional stage toward plaque psoriasis remains unresolved. Current cohorts are concentrated in the acute phase and rarely follow the same patients through conversion, remission, or recurrence, preventing direct reconstruction of TRM establishment and long-term disease evolution.

### 3.3 Generalized pustular psoriasis

Generalized pustular psoriasis (GPP) is a rare but potentially life-threatening inflammatory condition whose core mechanism is believed to involve excessive innate immune activation linked to IL-36 dysregulation (29, 43, 44, 48, 49, 54). Patients can rapidly develop widespread sterile pustules accompanied by high fever, leukocytosis, and multi-organ inflammatory responses, and GPP is therefore increasingly regarded as a systemic inflammatory disease (32, 43). Genetic studies have shown that variants in IL36RN, CARD14, AP1S3, and SERPINA3 are closely associated with the disease, with loss-of-function mutations in IL36RN being the most representative (45, 46). IL36RN encodes the IL-36 receptor antagonist (IL-36Ra); functional deficiency of this antagonist is associated with sustained IL-36 signaling and an impaired ability to properly terminate the inflammatory response. Upon stimulation, keratinocytes are thought to release large amounts of IL-36α, IL-36β, and IL-36γ, which in turn are predicted to induce the expression of CXCL1, CXCL8, G-CSF, and IL-6, and recruit neutrophils to the epidermis, ultimately giving rise to the characteristic Kogoj spongiform pustules (37, 44). At the same time, proteases released by neutrophils may further activate IL-36 precursors, potentially establishing an IL-36/neutrophil/IL-36 positive feedback loop, consistent with the concept of a disease process involving IL-36 pathway amplification (55, 56).

IL-36 dysregulation is central to GPP, but the relative contributions of IL-17, TNF, and type I interferon signaling remain incompletely defined. Moreover, many patients lack pathogenic IL36RN or related variants, indicating substantial genetic and environmental heterogeneity. A key mechanistic gap is explaining how genetically distinct cases converge on a similar neutrophilic inflammatory phenotype.

### 3.4 Palmoplantar pustulosis

Palmoplantar pustulosis (PPP) is a chronic, relapsing pustular disorder confined to the palms and soles, and its immunological features differ considerably from those of both GPP and PV (30, 54). In recent years, mounting evidence has supported the view that PPP may represent a distinct disease entity rather than a localized variant of GPP. The palmoplantar region is characterized by a high density of sweat gland ducts, neurovascular structures, and chronic mechanical stress, which together are thought to shape a unique local immune niche. Sweat gland duct epithelial cells are considered a likely site of inflammatory initiation, with activation of IL-17/IL-36-related pathways thought to trigger the inflammatory cascade (30, 37, 49). Single-cell studies have revealed a highly plastic T-cell state network in PPP: a subset of Th17 cells can transition toward a Th1-like phenotype, co-expressing IL17A, IFNG, and GM-CSF, thereby acquiring a more potent pro-inflammatory profile. Concurrently, keratinocytes and myeloid cells continuously release IL-36, CXCL8, and S100A8/S100A9, which are predicted to promote prolonged neutrophil retention and impede the resolution of inflammation. Consequently, the core pathogenic model of PPP has been extended from a predominant IL-17 abnormality to a framework of local microenvironment/keratinocyte/T-cell state remodeling.

PPP remains controversial because molecular overlap with GPP coexists with a distinctive palmoplantar microenvironment. Available datasets combine clinically heterogeneous cases and rarely include matched comparisons with GPP or PV, making it difficult to determine which T-cell and stromal programs are disease-specific. Direct cross-phenotype studies using harmonized palmoplantar sampling are needed to establish a reproducible molecular taxonomy.

### 3.5 Erythrodermic psoriasis

Erythrodermic psoriasis (EP) is one of the most severe subtypes of psoriasis, in which inflammation appears to breach the confines of the skin and progress to a state of systemic immune dysregulation (32, 51). Patients typically present with involvement of more than 90% of the body surface area, often accompanied by impaired thermoregulation, fluid and electrolyte disturbances, and metabolic abnormalities. Unlike PV, EP is characterized by the concomitant activation of Th1, Th2, and Th17 pathways, with marked elevations of IL-4, IL-13, IL-17A, IL-22, TNF-α, and IFN-γ, suggesting that the disease has evolved from a localized inflammatory process into a systemic inflammatory syndrome. Single-cell studies have shown that, in addition to Th17 cells, inflammatory monocytes, LAMP3^+^ dendritic cells, activated fibroblasts, and IL36G-high keratinocytes are all substantially expanded, and that they are spatially enriched in lesion-associated regions, consistent with a role in sustaining the systemic inflammatory state through complex intercellular communication networks (14, 15, 28, 40). EP may thus represent a terminal stage in which psoriasis shifts from chronic local inflammation to a state of systemic immune imbalance.

Because EP is rare and clinically severe, available transcriptomic cohorts are small and often lack matched non-lesional, resolving, or post-treatment samples. It therefore remains difficult to distinguish primary EP mechanisms from secondary responses to extensive systemic inflammation. Multicenter longitudinal sampling will be particularly important for resolving this distinction.

### 3.6 Psoriatic arthritis

**Figure 2:**
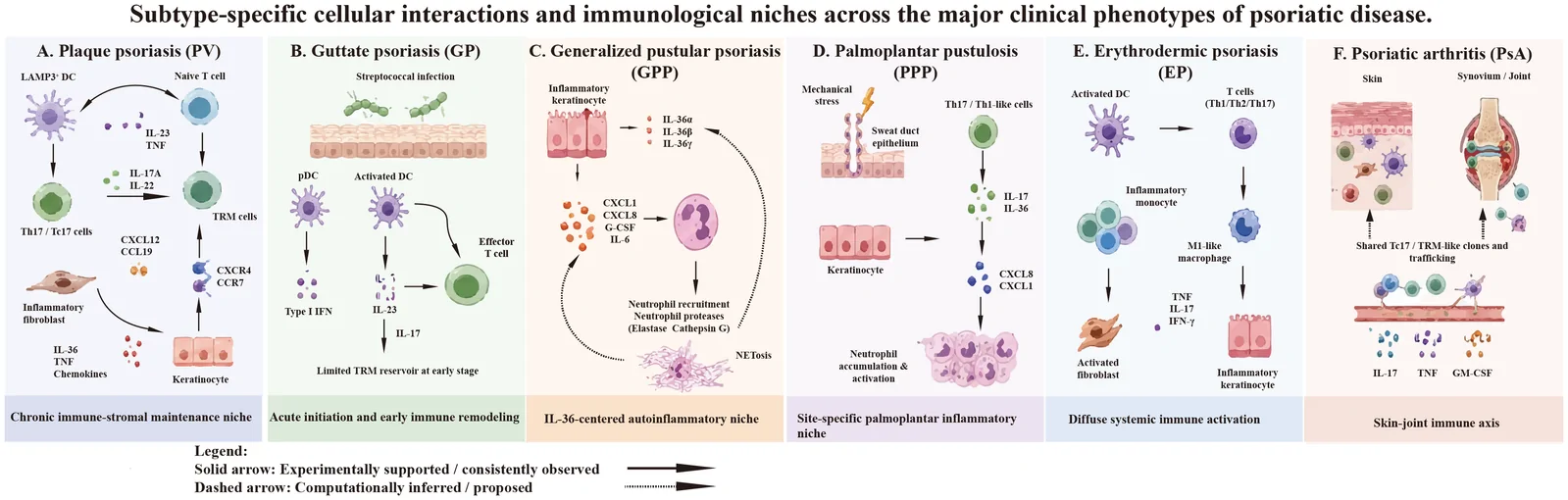
Subtype-specific cellular interactions and immunological niches across the major clinical phenotypes of psoriatic disease. The major clinical phenotypes of psoriatic disease are associated with partially overlapping but distinct cellular ecosystems and inflammatory programs. **(A)** Plaque psoriasis (PV) is characterized by a chronic immune-stromal maintenance niche involving LAMP3^+^ dendritic cells, Th17/Tc17 cells, tissue-resident memory T (TRM) cells, inflammatory fibroblasts, and keratinocytes. Major signaling programs include IL-23/TNF-mediated T-cell activation, IL-17A/IL-22-mediated keratinocyte responses, and the predicted CXCL12-CXCR4 and CCL19-CCR7 communication axes. **(B)** Guttate psoriasis (GP) represents an acute, frequently streptococcus-associated inflammatory state involving plasmacytoid dendritic-cell activation, type I interferon signaling, activated dendritic cells, and early effector T-cell responses, with a relatively limited TRM reservoir during the initial stage. **(C)** Generalized pustular psoriasis (GPP) is characterized by an IL-36-centered autoinflammatory niche in which inflammatory keratinocytes produce IL-36 cytokines and neutrophil-recruiting mediators, including CXCL1, CXCL8, G-CSF, and IL-6. Recruited neutrophils may further amplify IL-36 signaling through protease release and NETosis. **(D)** Palmoplantar pustulosis (PPP) is associated with a site-specific palmoplantar inflammatory niche shaped by mechanical stress, sweat duct epithelium, keratinocyte activation, plastic Th17/Th1-like cell states, IL-17/IL-36 signaling, and persistent neutrophil accumulation. **(E)** Erythrodermic psoriasis (EP) exhibits diffuse immune activation involving activated dendritic cells, inflammatory monocytes and macrophages, T cells, fibroblasts, and keratinocytes, accompanied by broad TNF-, IL-17-, and interferon-associated inflammatory programs. **(F)** Psoriatic arthritis (PsA) extends the psoriatic immune ecosystem beyond the skin through a proposed skin-joint axis involving shared Tc17/TRM-like clonotypes, circulating inflammatory mediators, and immune-cell trafficking between cutaneous and synovial compartments. Solid arrows indicate experimentally supported or consistently observed relationships, whereas dashed arrows indicate computationally inferred or proposed interactions requiring further functional validation.

Psoriatic arthritis (PsA) is one of the most important systemic complications of psoriasis, with approximately 20-30% of patients eventually developing joint involvement (33, 50, 57). PsA is increasingly recognized as a distinct clinical subtype of psoriatic disease that reflects extension along the skin-joint axis, rather than a wholly separate condition. The skin-joint axis theory proposes that persistent cutaneous inflammation may influence the joint microenvironment through circulating inflammatory mediators, immune cell trafficking, and shared antigen recognition. The IL-23/Th17 axis is thought to serve as an important bridge linking the skin and the joint: IL-17A, GM-CSF, and TNF-α released by Th17, Tc17, and TRM cells may act on synovial fibroblasts, osteoclasts, and endothelial cells, thereby contributing to inflammatory amplification and aberrant bone remodeling (35, 57, 58). Single-cell studies have further identified shared Tc17 and TRM-like cell clones across skin, peripheral blood, and synovial tissue, suggesting that immune cells are capable of trans-organ migration and may contribute to disease dissemination. In addition, synovium-specific inflammatory fibroblasts appear to be associated with a sustained inflammatory network involving immune cells via CXCL12, CCL2, and IL-6 (59). Collectively, these findings indicate that PsA illustrates a continuum in which psoriasis evolves from a localized skin disorder into a multi-organ immune-mediated disease, further underscoring that psoriasis is fundamentally a systemic inflammatory condition rather than a disorder confined to the skin (**Figure 2**).

Increasing evidence supports the concept of a skin-joint immune axis in PsA; nevertheless, the precise mechanisms underlying immune-cell migration between skin and synovium remain poorly understood. Although shared T-cell clonotypes have been identified across tissues, current findings do not establish direct migratory trajectories or causal relationships. Integrating single-cell transcriptomics with T-cell receptor lineage tracing and spatial analyses may help clarify how cutaneous inflammation evolves into systemic musculoskeletal disease.

## 4 The immune cell landscape in psoriasis: from classical cell types to subtype-specific states

With advances in scRNA-seq, ST, and multi-omics integration, psoriasis research is transitioning from an era centered on cellular composition to one increasingly defined by cell states and cellular ecosystems (9, 10). A growing body of evidence suggests that different psoriasis subtypes not only display distinct cellular compositions, but also harbor specific transcriptional states, spatial distribution patterns, and intercellular communication networks (28). Accordingly, the central question of the field has been gradually shifting from which cells are involved in disease pathogenesis to what functional states these cells adopt, how they interact, and how they may collectively contribute to disease persistence.

### 4.1 Innate immune cells: initiators and amplifiers of the inflammatory network in psoriasis

#### 4.1.1 Dendritic cells: a central hub connecting innate and adaptive immunity

Dendritic cells (DCs) are considered to serve both as an initiating node in the psoriatic immune network and as an important bridge linking innate and adaptive immunity (34, 60). The major populations currently recognized include plasmacytoid dendritic cells (pDCs), classical dendritic cells (cDCs), and the more recently identified DC3 subset. Early in the disease, following triggers such as infection, mechanical injury, or exposure to antimicrobial peptides, pDCs are thought to rapidly infiltrate the dermis and secrete IFN-α, which may initiate an antiviral-like inflammatory program. Subsequently, cDC2s are thought to become a major source of IL-23, TNF-α, and IL-12, promoting the expansion of Th17/Tc17 cells and sustaining chronic inflammation (36). Single-cell studies have further revealed a markedly expanded DC3 subset within psoriatic lesions; this population exhibits features of both monocytes and dendritic cells, with high expression of IL23A, TNF, IL1B, and FCN1, and is considered a potential link between innate immunity and chronic inflammation (14, 60). Spatial transcriptomic analyses have shown that LAMP3^+^ mature dendritic cells are predominantly located in the deep dermis and perivascular regions, where they are spatially enriched near Tc17 cells, TRM cells, and inflammatory fibroblasts, consistent with a role in long-term disease maintenance and tissue remodeling. It is worth noting that the composition of dendritic cell populations may differ substantially across psoriasis subtypes: guttate psoriasis is characterized by prominent pDC activation, whereas plaque psoriasis, erythrodermic psoriasis, and psoriatic arthritis tend to show enrichment of LAMP3^+^ mature dendritic cells, suggesting a staged process of immunological remodeling over the disease course.

Single-cell analyses have refined DC classification, but current labels may describe transient activation states rather than stable lineages. Differences in tissue dissociation, sequencing depth, and annotation contribute to inconsistent recovery of DC3 and LAMP3^+^ populations. Spatial localization combined with functional perturbation is needed to determine which subsets initiate inflammation and which primarily sustain established lesions.

#### 4.1.2 Neutrophils: from terminal effector cells to active contributors to inflammation

Neutrophils are the most representative effector cells in pustular psoriasis and are thought to be major contributors to acute inflammatory flares (43, 48). Although traditionally viewed primarily as contributors to sterile pustule formation, a growing body of evidence indicates that neutrophils themselves may be integral components of the network that sustains inflammation (61, 62). During the formation of neutrophil extracellular traps, neutrophils release DNA, histones, myeloperoxidase, and antimicrobial peptides, molecules that are predicted to activate dendritic cells and facilitate the cleavage of IL-36 precursors into their highly active forms, further amplifying the inflammatory response (63). In GPP, this is thought to give rise to the characteristic IL-36/neutrophil/IL-36 positive-feedback loop, whereas in PPP, it manifests as a state of persistent, low-level neutrophil activation (56). An increasing number of studies now suggest that neutrophils have moved beyond their assigned role as terminal effector cells and are better understood as active modulators that may help shape the immune microenvironment. Direct evidence for this modulatory role in human psoriasis tissue remains limited.

#### 4.1.3 Monocytes and macrophages: a bridge linking inflammation and tissue remodeling

Macrophages and monocytes are thought to serve as important amplifiers in sustaining the chronic inflammation of psoriasis. Single-cell studies have identified a predominant enrichment of CCR2^+^ inflammatory monocytes and SPP1^+^ macrophage subsets within lesional skin (14, 15, 40, 55). These cells continuously secrete TNF-α, IL-1β, IL-23, and IL-6, maintaining long-term activation of the Th17 inflammatory axis, while also being predicted to interact with fibroblasts through the CXCL12-CXCR4 and CCL2-CCR2 signaling axes. SPP1^+^ macrophages are considered a potential bridge linking the inflammatory response to tissue remodeling. In PsA, this subset has been implicated not only in synovial inflammation but also in aberrant bone remodeling, suggesting that it may serve as an important mediator in the extension of disease from the skin to the joints (33). This conclusion is supported mainly by cross-sectional scRNA-seq datasets; functional validation is still needed.

#### 4.1.4 Other innate immune cells

Additional innate immune cells implicated in psoriasis include mast cells, natural killer (NK) cells, and innate lymphoid cells (ILCs). Although they are not considered core drivers of psoriasis, they are thought to act as auxiliary amplifiers that contribute to local immune regulation. Mast cells can release histamine, TNF, and VEGF, and are spatially associated with neuroinflammation and vasodilation; NK cells may contribute to inflammatory amplification through the secretion of IFN-γ and TNF; and ILC3s produce IL-17 and IL-22, forming a parallel inflammatory pathway alongside Th17 cells that may help sustain the disease state (63–65). Despite their relatively low abundance, the role of these cells in shaping the local microenvironment is attracting increasing attention. The functional significance of these populations in psoriasis remains largely inferred from mouse models and *in vitro* studies.

### 4.2 Adaptive immune cells: from Th17-centric model to T-cell state heterogeneity

#### 4.2.1 Th17 and Tc17 cells: central drivers of disease persistence

Th17 and Tc17 cells have long been regarded as key pathogenic cell populations in psoriasis. Upon IL-23 stimulation, they produce IL-17A, IL-17F, IL-22, GM-CSF, and TNF, which are thought to promote aberrant keratinocyte proliferation and contribute to a self-perpetuating inflammatory amplification loop (35, 39). In certain disease stages or subtypes, the contribution of Tc17 cells may have been underestimated, and their role in disease maintenance and recurrence is now increasingly appreciated. Tc17 cells predominantly reside in the epidermis, exhibit a greater capacity for tissue residency, and have been more intimately linked to disease memory and post-treatment relapse (52). Accordingly, psoriasis research is progressively moving from a Th17-centric model toward a framework of T-cell state heterogeneity, in which T cells occupying distinct functional states collectively form the basis for long-term disease persistence (53).

#### 4.2.2 Th1 and Th22: parallel inflammatory branches

Th1 and Th22 cells are thought to represent important complementary components of the psoriatic inflammatory network (66, 67). Th1 cells mainly produce IFN-γ, which can promote dendritic cell activation, while Th22 cells secrete IL-22, thereby inducing epidermal thickening and impaired barrier function. Some studies have suggested that there may not be an absolute boundary among Th17, Th1, and Th22 cells; rather, they appear to form a continuous spectrum of functional states. Such T-cell plasticity is particularly evident in PPP and GPP, implying that the disease is probably not attributable to a single-cell population but is instead associated with a dynamically evolving T-cell network. This inference is based on cross-sectional transcriptomic profiling; functional lineage-tracing studies are lacking (30, 37, 49, 67).

#### 4.2.3 Regulatory T cells: from immunosuppression to functional imbalance

Unlike classical autoimmune diseases, psoriasis is not typically associated with a marked reduction in Treg numbers; rather, the principal defect appears to lie in functional imbalance (68). Within a chronic inflammatory milieu, a subset of Tregs may undergo inflammatory reprogramming, with some converting to FOXP3^+^ IL-17A^+^ double-positive cells and losing suppressive capacity. IL-17 and IL-23 inhibition has been associated with partial restoration of Treg phenotypes, but existing treatment studies rarely combine longitudinal profiling with direct suppressive-function assays.

#### 4.2.4 Tissue-resident memory T cells: core mediators of disease memory

Tissue-resident memory T (TRM) cells represent one of the most significant discoveries in psoriasis research in recent years and are considered a key cellular substrate of disease memory (40, 52). In psoriasis, TRM cells are predominantly CD8^+^, characteristically express CD69, CD103, and CXCR6, and continually produce IL-17A, IL-17F, IL-22, and IFN-γ. A lineage continuity appears to exist between Tc17 and TRM cells: some Tc17 cells may gradually acquire a tissue-resident phenotype in the course of chronic inflammation, becoming an important source for the long-term maintenance of the disease (39). These cells persist at the epidermal-dermal junction even after lesions have resolved clinically. When re-exposed to triggers such as infection, mechanical stress, or treatment withdrawal, TRM cells are thought to rapidly reactivate the local inflammatory response. TRM cells are therefore widely regarded as an important factor underlying disease recurrence and local relapse in psoriasis, and as a key therapeutic target for achieving long-term remission.

Despite accumulating evidence supporting TRM cells as key mediators of disease memory, several unresolved questions remain. In particular, the developmental relationship between circulating effector T cells, Tc17 cells, and long-lived TRM populations has not been fully established. Moreover, although TRM persistence is strongly associated with disease recurrence, the molecular mechanisms governing their long-term survival within resolved lesions remain incompletely understood. Longitudinal lineage-tracing and functional studies are therefore needed to determine whether TRM cells are true drivers of relapse or markers of persistent tissue inflammation. These studies should also clarify how TRM cells interact with stromal and innate immune compartments to maintain residual inflammatory niches.

#### 4.2.5 Epigenetic regulation of T-cell plasticity and disease memory

Epigenetic regulation provides an additional layer for understanding how transient inflammatory signals may be converted into relatively persistent T-cell states in psoriasis. Whereas scRNA-seq captures the transcriptional activity of individual cells at a specific time point, chromatin accessibility reflects the regulatory potential of promoters, enhancers, and transcription-factor-binding regions. Integrating transcriptomic and epigenomic information may therefore help distinguish short-lived activation responses from more stable pathogenic programs that contribute to T-cell plasticity, tissue persistence, and disease recurrence.

ATAC-seq profiling of psoriasis-associated T-cell populations has identified altered chromatin-accessibility landscapes across naïve and memory T cells, including Th1, Th17, Th1/17, and regulatory T-cell populations. Changes in accessible chromatin regions were associated with corresponding differences in gene expression, particularly in memory helper T cells and Tregs, suggesting that chromatin remodeling may contribute to the maintenance of inflammatory transcriptional programs and impaired immune regulation in psoriasis (56). These findings extend the conventional cytokine-centered model by indicating that pathogenic T-cell behavior may also depend on the accessibility of regulatory elements that determine how cells respond to IL-23, IL-17-related signals, interferons, and other components of the inflammatory microenvironment.

Epigenetic remodeling may also provide a possible framework for interpreting the functional plasticity observed among Th17, Th1-like, Tc17, Treg, and tissue-resident memory T-cell states. Chronic exposure to inflammatory cytokines could alter chromatin accessibility and thereby increase the responsiveness of T cells to subsequent stimulation. Such regulatory changes might facilitate the acquisition of mixed inflammatory phenotypes, including IL-17- and IFN-γ-associated programs, or contribute to the impaired suppressive function of Tregs. Similarly, persistent epigenetic programs in TRM cells could theoretically support their long-term survival and rapid reactivation in clinically resolved lesions, thereby contributing to local disease memory and recurrence (52, 68).

However, current evidence remains largely associative. Differences in chromatin accessibility do not by themselves establish that a specific regulatory element causes T-cell conversion, persistence, or relapse. Moreover, most available studies have examined peripheral or cross-sectional samples rather than longitudinally paired active, resolved, and recurrent lesions. Future studies integrating scATAC-seq, scRNA-seq, T-cell receptor clonotype analysis, spatial transcriptomics, and functional perturbation will be required to reconstruct the regulatory trajectories linking circulating effector cells, pathogenic Tc17/Th17 states, dysfunctional Tregs, and long-lived TRM populations. Such approaches may clarify whether epigenetic remodeling represents a reversible consequence of inflammation or a stable molecular substrate underlying disease memory.

### 4.3 Stromal and epithelial cells: from tissue scaffolds to immunological niche architects

#### 4.3.1 Keratinocytes: the major inflammatory amplifiers of the disease

Keratinocytes have long been considered merely target cells of the inflammatory response; however, a growing body of evidence now points to their role as important regulators of the psoriatic immune network (14, 38). Upon stimulation with IL-17A and TNF-α, keratinocytes are thought to upregulate a broad array of pro-inflammatory molecules, including CXCL1, CXCL8, CCL20, S100A7, S100A8/S100A9, and IL36G, which are predicted to collectively recruit Th17 cells, neutrophils, and dendritic cells, potentially forming a self-sustaining inflammatory circuit. Single-cell studies have further revealed marked state heterogeneity among keratinocytes, which can be parsed into proliferative, inflammatory, interferon-responsive, and stress-associated functional states, and undergo dynamic transitions across different stages of the disease (69).

#### 4.3.2 Fibroblasts: from structural cells to immunoregulatory cells

Fibroblasts have undergone a conceptual reclassification from tissue scaffold cells to immunoregulatory cells (14, 55, 70, 71). Single-cell studies have identified a population of inflammatory SFRP2^+^ fibroblasts within psoriatic lesions. Beyond expressing classical matrix-associated genes such as COL1A1, COL3A1, DCN, and LUM, these fibroblasts also exhibit high expression of CCL13, CCL19, CXCL12, and CTSS, thus displaying a dual functionality that encompasses both matrix remodeling and immune regulation. The CXCL12-CXCR4 signaling axis has been associated with macrophage recruitment, CCL19 with T-cell migration, and CTSS with antigen processing and inflammatory amplification (14, 15, 71). These findings suggest that fibroblasts, rather than being passive structural bystanders, may actively participate in sculpting the immune microenvironment.

Fibroblasts have emerged as active regulators of the psoriatic microenvironment rather than passive structural cells. However, the functional diversity of fibroblast subsets remains incompletely defined, and currently available classification schemes differ considerably among studies because of variations in analytical pipelines and marker selection. Whether inflammatory fibroblasts represent stable cell populations or reversible activation states also remains uncertain. Resolving these questions will be essential before fibroblast-targeted therapeutic strategies can be translated into clinical practice.

## 5 Cell-cell communication networks in psoriasis

Advances in single-cell sequencing and spatial transcriptomics are propelling psoriasis research from a single-cell pathogenicity paradigm toward a multi-cellular interactome paradigm (9, 10, 14, 15, 28, 40). Current evidence suggests that the initiation and persistence of psoriasis are not attributable to a single-cell type or cytokine in isolation, but are instead shaped by dynamic, plastic, and spatially organized communication networks among keratinocytes, immune cells, fibroblasts, and myeloid cells. The disease may be understood, in essence, as a self-sustaining inflammatory ecosystem whose formation and maintenance are critically dependent on cell-state transitions, ligand-receptor interactions, and local tissue microenvironment remodeling (72). These spatially restricted cellular behaviors undergo dynamic rewiring during disease progression, creating localized inflammatory loops. A comparative overview of subtype-specific spatial niches and their dominant communication mechanisms is provided in **Table 3**.

**Table 3:** Multidimensional pathological ecosystems and therapeutic reprogramming across psoriasis subtypes.

Study	Psoriasis subtype	Key immunopathological features of the cellular ecosystem	Spatial niche architecture	Core communication mechanisms	Disease maintenance mechanisms	Therapeutic reprogramming strategies
Ma F, et al. (2023) (14)	Plaque psoriasis	CD8+ Tc17 cells appear to be a major source of IL-17A; SFRP2+ fibroblasts, LAMP3+ cDC2A, and CD16+ DCs are substantially expanded.	The suprabasal epidermis seems to serve as a hub for IL-17/TNF/IL-36-driven inflammation; SFRP2+ fibroblasts and myeloid cells are enriched in the papillary dermis.	CCL19-CCR7, CXCL12-CXCR4, CCL13-CCR2, and IL-36G-IL-36R may constitute a core communication network.	SFRP2+ fibroblasts might activate IL-36G through cathepsin S, potentially establishing an IL-36-IL-17/TNF positive-feedback loop.	Targeting IL-36G/IL-36R or inhibiting CCL19/CCR7 could disrupt the inflammatory network and may promote remission.
Gniadecki R, et al. (2023) (40)	Plaque psoriasis (spatial niche-based view)	Innate immunity appears to predominate; a superficial dermal LM2 (lympho-macrophage) niche is formed, and an LRRC15+ fibroblast subset is identified in the deep dermis.	The epidermis seems to be characterized primarily by IL-1/IL-36-driven inflammation; the papillary dermis shows high IL-32/IFN expression; the deep dermis forms an independent stromal niche.	The IL-18-IL-32 axis may drive dermal inflammation, and a chemokine network likely facilitates immune cell aggregation.	TRM cells together with persistent TGF-β signals might create a “molecular residual memory” that could mediate recurrence.	IL-23 inhibitors may remodel inflammatory niches; IL-32 and IL-1 family members might represent novel therapeutic targets.
Francis L, et al. (2024) (15)	Severe plaque psoriasis	The IL-23-Th17/Tc17 axis is thought to drive the disease; lesions are enriched in DC3, mregDC, TEMRA T cells, and pro-inflammatory fibroblasts.	A WNT5A+/IL24+ fibroblast niche appears to form in the upper dermis, in close proximity to proliferating keratinocytes.	IL-17/TNF signals may induce WNT5A+/IL24+ fibroblasts, and IL-24 could further activate keratinocytes.	A Th17-fibroblast-keratinocyte feed-forward loop is proposed to participate in both disease maintenance and relapse-associated memory.	IL-23 or IL-17 inhibition might reprogram WNT5A+/IL24+ fibroblasts toward a homeostatic COMP+ state.
Schäbitz A, et al. (2022) (28)	Classical type-3 immunity-mediated psoriasis	Core pathogenic factors such as IL-17A exhibit very low transcript abundance yet appear to exert potent pathogenic effects.	Inflammation tends to occur in localized epidermal clusters, with close spatial proximity between pathogenic factors and responding cells.	Even trace amounts of IL-17A may be sufficient to trigger a large-scale downstream inflammatory cascade.	Spatial amplification effects likely enable the local microenvironment to sustain a persistent inflammatory state.	The strategy may need to shift from broad-spectrum anti-inflammatory approaches toward the precise blockade of low-abundance, high-potency pathogenic networks.

This table compares anatomically restricted cellular niches, dominant ligand–receptor axes, proposed maintenance mechanisms, and potential therapeutic strategies across major phenotypes. The pathways shown provide a comparative framework for interpreting immune–stromal organization and residual tissue memory.

Computational ligand-receptor analysis has expanded the range of candidate communication circuits in psoriasis, but its results require a distinct evidentiary framework. The assumptions, confounders, and validation strategies for these analyses are discussed in Section 4.5.

### 5.1 Keratinocyte-immune cell interactions

The bidirectional crosstalk between keratinocytes and immune cells is thought to constitute an important mechanism contributing to the persistence of chronic inflammation in psoriasis and provides the cellular basis for the sustained activation of the classical IL-23/IL-17/TNF inflammatory axis (38, 69). Dendritic cells and myeloid cells, activated by genetic predisposition, mechanical stimuli, or microbe-associated signals, secrete IL-23, TNF-α, and other factors that are thought to promote the expansion and maintain the pathogenic phenotype of Th17 and Tc17 cells (34–36). Activated Th17/Tc17 cells in turn produce effector molecules such as IL-17A, IL-17F, IL-22, and GM-CSF, which act on keratinocytes. Under the synergistic stimulation of IL-17A and TNF-α, keratinocytes are thought to shift from passive targets to active inflammatory amplifiers, markedly upregulating chemokines, antimicrobial peptides, and inflammatory mediators including CXCL1, CXCL8, CCL20, S100A7, S100A8, S100A9, and IL36G (38). Among these, CXCL1 and CXCL8 are predicted to primarily recruit and activate neutrophils; CCL20 selectively attracts CCR6^+^ Th17/Tc17 cells back into the lesion; and S100 family proteins not only participate in antimicrobial defense but may also act as damage-associated molecular patterns to further amplify the local inflammatory response. This signaling cascade may establish a self-sustaining positive-feedback loop. IL-23 and TNF promote Th17/Tc17 activation, whereas IL-17A and IL-17F stimulate keratinocytes to release chemokines and inflammatory mediators. These mediators subsequently recruit additional immune cells and may sustain local IL-23 production. As inflammation persists, keratinocyte-derived IL-36 is thought to further bridge innate and adaptive immunity, potentially transforming the originally linear IL-23/IL-17 pathway into a multi-tiered amplification network.

### 5.2 Fibroblast immune cell interactions

Fibroblasts have emerged as an important new cell population in psoriasis research (14). Single-cell transcriptomic studies have shown that fibroblasts are not merely stromal cells maintaining tissue architecture, but rather functionally versatile cells with notable immunoregulatory capabilities (71). In plaque psoriasis, single-cell transcriptomic analyses have identified an inflammatory fibroblast population characterized by SFRP2, WNT5A, IL24, CCL19, CXCL12, and CTSS expression. Ligand-receptor inference and spatial colocalization analyses suggest that these fibroblasts may communicate with Tc17 cells, dendritic cells, macrophages, and keratinocytes through several signaling axes. CXCL12-CXCR4 signaling may contribute to the recruitment or retention of CXCR4^+^ immune cells, whereas CCL19-CCR7 signaling may influence the localization of dendritic cells and memory T cells. IL-24-related signaling has also been predicted to activate inflammatory transcriptional programs in keratinocytes, including S100A7, S100A8, S100A9, and CXCL8 expression.

These observations support a model in which inflammatory fibroblasts act as potential organizers of the local immune microenvironment rather than passive structural cells. However, these interactions are inferred primarily from transcript co-expression and spatial proximity. Their directionality, functional activity, and causal contribution to psoriasis therefore require validation using protein-level localization, co-culture systems, and targeted perturbation experiments.

### 5.3 Myeloid-epithelial and myeloid-stromal circuits

Myeloid cells are thought to serve as an important bridge linking innate immunity with the tissue microenvironment, and may perform dual functions of signal integration and inflammatory amplification within the psoriatic inflammatory network (14, 40). Dendritic cells, monocytes, and macrophages can continuously secrete IL-1β, IL-23, TNF, and CCL2, promoting T-cell expansion and activating keratinocytes; conversely, keratinocytes and fibroblasts release chemokines such as CXCL1, CXCL8, CCL19, and CXCL12, further recruiting myeloid cells into the lesion and establishing a sustained inflammatory circuit (34, 36, 73). This network appears to manifest differently across subtypes: in GPP, it is primarily reflected in the IL-36γ/neutrophil/IL-36γ positive-feedback loop; in PV and PsA, it is more typically represented by the LAMP3^+^ DC/Tc17/SFRP2^+^ fibroblast chronic maintenance network (29, 30). These observations imply that myeloid cells may constitute a shared inflammatory scaffold across psoriasis subtypes, whereas distinct clinical phenotypes arise from the remodeling of specific cell states and tissue microenvironments. This inference is based on cross-sectional comparisons; experimental confirmation is lacking.

### 5.4 Spatially restricted pathological niches

**Figure 3:**
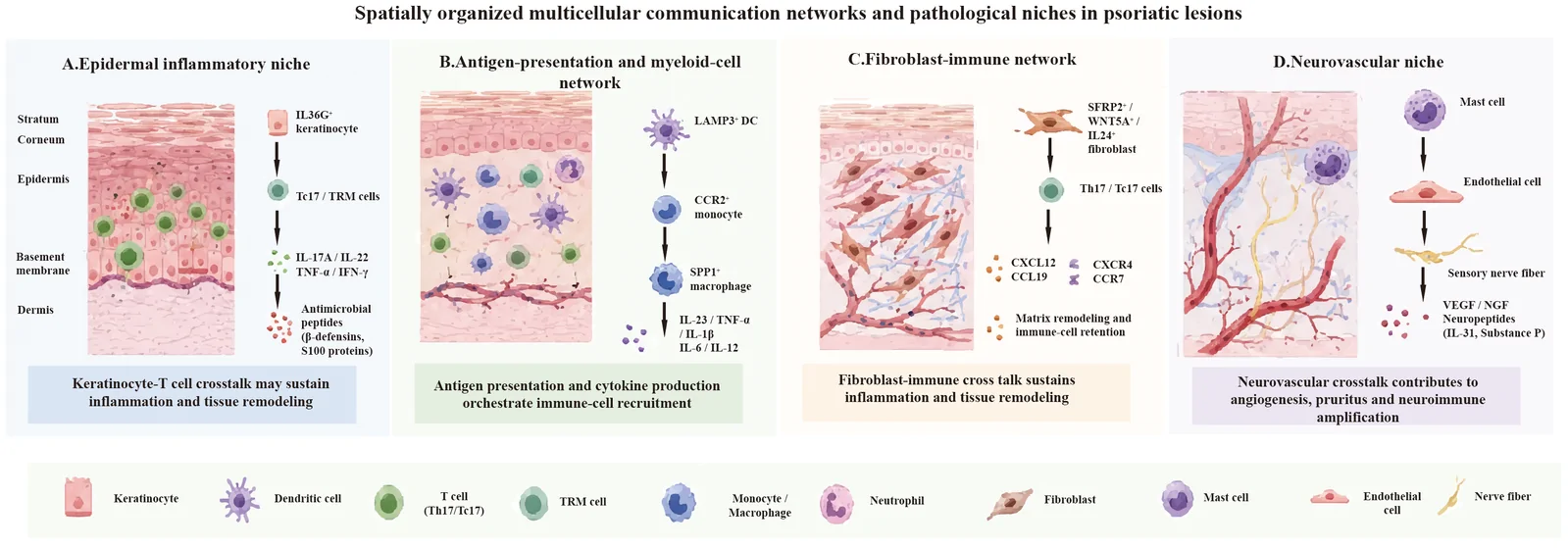
Spatially organized multicellular niches and intercellular communication networks in psoriatic skin. Psoriatic lesions can be conceptualized as interconnected epidermal, dermal immune, stromal, and neurovascular niches. **(A)** The epidermal inflammatory niche contains IL36G-high keratinocytes and epidermal Tc17/TRM cells. IL-17A, IL-22, TNF, and interferon-associated signals promote keratinocyte inflammatory activation and the production of antimicrobial peptides, including β-defensins and S100 proteins, thereby contributing to epidermal hyperplasia and barrier dysfunction. **(B)** The dermal antigen-presenting and myeloid niche contains LAMP3^+^ dendritic cells, CCR2^+^ inflammatory monocytes, SPP1^+^ macrophages, and additional infiltrating immune populations. These cells are associated with antigen presentation, production of IL-23, TNF, IL-1β, IL-6, and IL-12, and recruitment or activation of pathogenic T-cell populations. **(C)** The fibroblast-immune stromal niche is enriched in inflammatory fibroblasts expressing SFRP2, WNT5A, IL24, CXCL12, and CCL19. Computational and spatial analyses suggest that these fibroblasts may communicate with Th17/Tc17 cells and other immune populations through pathways including CXCL12-CXCR4 and CCL19-CCR7, potentially contributing to immune-cell retention, matrix remodeling, and maintenance of local inflammation. **(D)** The neurovascular niche consists of mast cells, vascular endothelial cells, and sensory nerve fibers. VEGF, NGF, IL-31, substance P, and other neuroimmune mediators may contribute to vascular remodeling, pruritus, and neurogenic amplification of inflammation. These niches are spatially interconnected and collectively form the psoriatic tissue immune ecosystem. The figure is schematic and does not represent quantitative cellular abundance or establish causal interactions.

Spatial transcriptomic data suggest that psoriasis is not composed of randomly distributed inflammatory cells, but rather comprises multiple pathological niches with discernible boundaries (41). Distinct cell populations, through spatial proximity and local ligand-receptor interactions, form stable functional units that are associated with disease initiation, maintenance, and expansion. Representative niches include: the epidermal inflammatory niche, composed of IL36G^+^ keratinocytes, Tc17 cells, and TRM cells, which is associated with local inflammatory maintenance and disease memory; the dermal immune niche, formed by LAMP3^+^ dendritic cells, CCR2^+^ monocytes, and SPP1^+^ macrophages, which is predicted to handle inflammatory signal integration and immune recruitment; the stromal niche, centered on SFRP2^+^/WNT5A^+^/IL24^+^ inflammatory fibroblasts, which coordinates matrix remodeling and immune regulation; and the neurovascular niche, jointly constituted by mast cells, vascular endothelial cells, and nerve-associated cells, which is implicated in pruritus, vasodilation, and inflammatory amplification. These niches are not isolated from one another, but rather form a cross-hierarchical communication network through chemokines, cytokines, and migratory signals, collectively constituting the spatially organized immune ecosystem that is characteristic of psoriasis (40) (**Figure 3**).

### 5.5 Computational inference of cell-cell communication: strengths, limitations, and functional validation

Computational analysis of cell-cell communication has become an important extension of single-cell and spatial transcriptomic studies. Tools such as CellChat, CellPhoneDB, NicheNet, and related frameworks infer potential communication events by matching ligand expression in one cell population with receptor expression in another (72, 74–76). These approaches can prioritize candidate signaling pathways, compare communication patterns across disease states, and generate mechanistic hypotheses concerning the organization of psoriatic immune niches (76). However, their results should not be interpreted as direct evidence of physical or functional cellular interactions.

First, ligand and receptor transcript abundance does not necessarily reflect protein abundance, secretion, receptor accessibility, post-translational modification, or downstream pathway activation. A ligand-receptor pair may therefore be transcriptionally detectable without being biologically active. Conversely, transcript dropout and limited detection sensitivity may cause biologically relevant interactions to be missed, particularly when ligands are transiently expressed or produced by rare cell populations. Second, computational predictions depend heavily on the reference ligand-receptor database and the analytical algorithm used. Different tools may use distinct interaction databases, scoring systems, statistical assumptions, and definitions of sender and receiver populations. Consequently, the same dataset may yield different communication networks when analyzed using alternative pipelines. Predictions are also influenced by clustering resolution and cell-type annotation. Broad cell clusters may conceal communication involving rare pathogenic states, whereas excessive subclustering may produce unstable or poorly reproducible interactions. Third, transcriptomic co-expression alone does not establish spatial proximity, directionality, or causality. Conventional scRNA-seq data do not retain the original positions of cells within tissue. Thus, predicted sender and receiver populations may not be located sufficiently close to interact *in vivo*. Spatial transcriptomic data can strengthen these predictions by demonstrating colocalization or neighborhood enrichment, but spatial proximity still does not prove that a ligand is secreted, binds its receptor, or produces a functional response. Similarly, inferred communication strength represents a computational score rather than a direct measurement of cytokine concentration, signaling flux, or biological effect.

Changes in cellular composition may further confound comparisons between disease states or treatment time points. For example, an apparent reduction in overall communication after therapy may reflect decreased abundance of inflammatory cell populations rather than selective disruption of a specific signaling pathway. Therefore, CellChat-derived interaction numbers or aggregate communication scores should not be equated directly with biological network activity. Comparisons should account for cell abundance, sequencing depth, sample size, and patient-level variability, and should preferably be reproduced using more than one computational method or independent cohort.

Functional validation is therefore required before computationally inferred communication pathways can be regarded as established pathogenic mechanisms. Multiplex immunofluorescence, RNA *in situ* hybridization, imaging mass cytometry, and spatial proteomics can determine whether the proposed sender and receiver populations are spatially adjacent and whether the corresponding ligands and receptors are present at the protein level. Co-culture systems incorporating keratinocytes, fibroblasts, dendritic cells, macrophages, and T cells can test whether a candidate cell population induces a predicted transcriptional or inflammatory response in another population. Neutralizing antibodies, receptor antagonists, gene silencing, CRISPR-based perturbation, and pharmacological inhibition can further determine whether disruption of a candidate signaling axis alters the predicted cellular response. Three-dimensional skin models, organotypic cultures, and *in vivo* psoriasis models may then be used to evaluate the relevance of these interactions within a more physiologically representative tissue environment.

Accordingly, computational cell-cell communication analysis should be viewed primarily as a hypothesis-generating and prioritization framework. Confidence is greatest when an interaction is consistently identified across independent datasets, supported by spatial colocalization, confirmed at the protein level, and functionally disrupted through targeted experimental intervention. Integrating these complementary levels of evidence will be essential for distinguishing reproducible pathogenic communication circuits from dataset-specific computational predictions.

## 6 Single-cell and spatial transcriptomic evidence across psoriasis subtypes

Single-cell sequencing and spatial transcriptomic technologies have become core tools for dissecting the heterogeneity of psoriasis. In contrast to the tissue-averaged signals provided by bulk RNA-seq, these approaches can simultaneously reveal cellular composition, state transitions, cell-cell interactions, and spatial architecture, thereby reshaping our understanding of psoriasis pathogenesis (8–10). Representative single-cell and spatial transcriptomic studies in psoriasis are summarized in **Table 4**.

**Table 4:** Representative single-cell and spatial transcriptomic studies in psoriasis.

Study	Psoriasis subtypes	Omics platform	Biological samples	Major findings
Ma F, et al. (2023) (14)	Plaque Psoriasis	scRNA-seq, Spatial RNA-seq	Human psoriatic lesional and epidermal tissue	1. Demonstrated that the stratum spinosum can amplify IL-17A/TNF inflammatory responses through IL-36, even in the absence of neutrophils. 2. Identified a core population of pro-inflammatory SFRP2+ fibroblasts that appear to engage in spatial communication with CCR2+ myeloid cells, CCR7+LAMP3+ DCs, and CD8+ Tc17 cells via the secretion of CCL13, CCL19, and CXCL12. 3. Found that this subset highly expresses cathepsin S, which may activate keratinocyte-derived IL-36G and establish an endogenous positive-feedback inflammatory loop.
Gniadecki R, et al. (2023) (40)	Plaque Psoriasis	Spatial Transcriptomics (10x Visium)	Human psoriatic lesional biopsies before and after treatment	1. Identified three spatially distinct inflammatory niches in psoriatic lesions: epidermal, superficial dermal, and deep dermal. 2. Observed that the superficial dermis forms a “lympho-macrophage” microenvironment centered on memory T cells and inflammatory macrophages, while an independent LRRC15+ fibroblast subset with cancer-associated fibroblast-like features was identified in the deep dermis. 3. Epidermal inflammation was found to be driven primarily by the IL-1/IL-36 pathway, with relatively low transcript abundance of classical IL-23/IL-17/TNF factors.
Francis L, et al. (2024) (15)	Plaque Psoriasis	Longitudinal scRNA-seq	Full-thickness human skin biopsies before and after IL-23 inhibitor treatment	1. Longitudinal single-cell analysis indicated that fibroblasts and myeloid cells are among the earliest responders to IL-23 inhibition. 2. Characterized a lesion-specific WNT5A+/IL24+ inflammatory fibroblast subset that can respond to TNF and IL-17 signaling and promote keratinocyte inflammation. 3. This subset declined rapidly during early treatment, accompanied by a concurrent restoration of epidermal inflammation and the vascular microenvironment.
Schäbitz A, et al. (2022) (28)	Psoriasis	Spatial Transcriptomics, scRNA-seq, Bulk RNA-seq	Full-thickness skin biopsies from lesional and non-lesional skin	1. Found that the transcript abundance of core pathogenic factors such as IL17A is very low, yet exhibits highly specific spatial distribution. 2. Small amounts of cytokines were shown to be sufficient to induce large-scale downstream inflammatory amplification, driving keratinocyte expression of pathogenic molecules such as IL19, CXCL8, and IL36G. 3. Provided evidence that spatial transcriptomics can resolve the “immune cell signal-tissue cell response” inflammatory network *in situ*.
Zhou Y, et al. (2022) (41)	Psoriasis	CyTOF (Mass Cytometry)	Full-thickness skin biopsies from lesional and non-lesional skin	1. Provided evidence that the epidermal immune microenvironment plays a dominant role in the initiation and progression of psoriasis. 2. Found that, during early disease, CD1c+CD11b+ cDC2s migrate from the dermis to the epidermis, replacing homeostatic Langerhans cells and initiating the inflammatory response. 3. With disease progression, CD207+CD11c Langerhans cells and CD5+ T cells accumulated, jointly maintaining a chronic epidermal inflammatory network.

This table provides a chronological synthesis of pioneering single-cell RNA sequencing (scRNA-seq) and spatial transcriptomics (ST) datasets. It traces the evolution of multi-omics platforms from droplet-based transcriptomic profiling to *in situ* tissue deconvolution—and highlights their landmark contributions to mapping cell-state heterogeneity and identifying rare pathological subsets. Particular emphasis is placed on studies that incorporate cross-platform validation and longitudinally tracked clinical cohorts, which collectively offer a valuable framework for resolving tissue-level architectural reorganization during both active inflammation and targeted therapeutic intervention.

### 6.1 Contributions of single-cell studies

The most important contribution of single-cell studies has arguably been to reveal the extensive cell-state heterogeneity that exists in psoriasis (14). Keratinocytes can be parsed into proliferative, inflammatory, and interferon-responsive states; fibroblasts can be divided into homeostatic and inflammatory subsets; myeloid cells display a spectrum of maturation and activation; and the T-cell compartment has been expanded from the traditional Th17-centric model to a complex network in which Tc17, TRM, and pathogenic Th17 cells act in concert. Computational analyses of single-cell transcriptomic data have predicted extensive ligand-receptor communication networks, thereby generating new hypotheses regarding the organization of pathogenic cell states in psoriasis.

### 6.2 Spatial transcriptomics: resolving lesion borders and inflammatory expansion fronts

The core value of spatial transcriptomics lies in its ability to preserve tissue topology, thereby providing direct evidence for disease evolution *in situ* (10, 14, 15, 19, 20, 26, 28, 40, 77). Using 10x Visium, high-resolution spatial transcriptomics, and single-cell imaging techniques, researchers have been able to identify lesional borders, inflammatory expansion fronts, and resolving regions within intact tissue contexts, and to construct continuous dynamic maps of the transition from normal skin to active psoriatic lesions. These *in situ* observations have provided support for the spatial pathological models proposed by single-cell communication analyses. The inflammatory expansion front appears not to be driven by a single-cell population, but rather by a highly organized microenvironment formed by inflammatory keratinocytes, myeloid cells, and stromal cells within a defined territory, which is associated with the spread of lesions into the surrounding normal skin through continuous signal propagation. The lesion border is not a static anatomical structure; instead, it represents a transcriptionally active, dynamic transition zone where cell-state conversions, chemokine signaling, and inflammatory amplification occur simultaneously, linking normal skin and established lesional tissue.

### 6.3 Longitudinal treatment studies as disease resolution models

Longitudinal single-cell studies have emerged as an important model for dissecting the mechanisms of disease resolution. Unlike traditional treatment studies that focus primarily on PASI scores, single-cell technologies allow dynamic tracking of the temporal sequence of cell-state changes following therapy (15). In some longitudinal cohorts, it has been observed that after IL-23 blockade, the earliest transcriptional remodeling occurs not in T cells, but rather in inflammatory fibroblasts and myeloid cells; by day 14, keratinocyte inflammatory programs are markedly downregulated, and the activity of Tc17 and TRM cells gradually attenuates thereafter. CellChat analysis predicted a reduction in the number and aggregate strength of ligand-receptor interactions after treatment. However, these communication scores represent computational estimates based on transcript expression and do not directly measure functional intercellular signaling (15, 72, 75). Consequently, therapeutic evaluation may need to move from a sole focus on inflammatory load toward assessing the restoration of cell states and communication networks. Evidence remains limited by small cohort size and lack of functional validation.

### 6.4 Subtype-specific cellular ecosystems

Different psoriasis subtypes appear to display distinct cellular ecosystems (54). PV is characterized primarily by the IL-23/IL-17 axis, with Tc17, TRM, LAMP3^+^ DCs, and inflammatory fibroblasts contributing to a chronic maintenance network; GPP exhibits an acute neutrophil–keratinocyte niche centered on IL-36 signaling; and PPP combines palmoplantar tissue specialization with T-cell plasticity and persistent neutrophilic inflammation. Direct cross-subtype studies using matched platforms and anatomical sampling remain scarce, so these distinctions should be regarded as comparative working models rather than fixed taxonomies.

## 7 Dynamic cellular remodeling during disease initiation, progression, and recurrence

Psoriasis is typically triggered by external factors such as infection, mechanical stress, psychological distress, drug exposure, or metabolic abnormalities (31, 34, 42, 78). In the early phase, plasmacytoid dendritic cells and myeloid cells are thought to be activated, releasing IFN-α, TNF-α, and IL-23, which may initiate the innate immune response and promote the formation of the Th17 axis (69). Recent studies have indicated that disease onset is not solely a process of immune cell activation, but also the beginning of tissue microenvironment remodeling; keratinocytes, fibroblasts, and vascular endothelial cells are thought to participate in signal propagation from the earliest stages, collectively shaping the subsequent chronic inflammatory network. Cross-sectional single-cell and spatial transcriptomic studies suggest that progressive psoriatic lesions are associated with increasingly coordinated inflammatory programs among immune cells and tissue-resident populations. Dendritic cells and myeloid cells express IL-23- and TNF-associated programs; Th17/Tc17 cells exhibit IL-17A- and IL-22-related activity; keratinocytes upregulate chemokines and antimicrobial peptides; and inflammatory fibroblasts are predicted to contribute to immune-cell recruitment and matrix remodeling. Collectively, these observations support a proposed transition from an acute inflammatory response to a more stably organized inflammatory ecosystem. However, because most available studies are cross-sectional, the temporal sequence and causal hierarchy of these cellular changes have not yet been established.

Even after clinical resolution of lesions, residual inflammatory niches may be retained in the local tissue (14, 15, 40, 52, 53). Tissue-resident memory T cells can persist long-term at the epidermal-dermal junction, and inflammatory fibroblasts may maintain a memory-like state (79). Upon re-exposure to triggers such as infection, mechanical stress, or treatment withdrawal, these cells are predicted to rapidly re-initiate inflammatory programs and precipitate recurrence. Therefore, future therapeutic goals may need to extend beyond clinical remission and aim toward the comprehensive remodeling of pathological niches. Evidence for the memory-like fibroblast state remains limited and requires further validation. Identifying and validating these highly plastic cell states is crucial for translating descriptive omics findings into targeted precision therapies. Consensus annotation markers and recommended validation markers for major pathogenic cell states are summarized in **Table 5**. While these single-cell and spatial omics studies have substantially advanced our understanding of dynamic immune-cell transitions, most currently proposed cellular interactions and differentiation trajectories remain computationally inferred rather than experimentally validated. Therefore, complementary functional approaches are required to establish causal relationships between immune-cell dynamics and disease progression. *In vitro* co-culture systems incorporating keratinocytes, fibroblasts, dendritic cells, and T cells can validate predicted cytokine circuits and ligand-receptor interactions, whereas three-dimensional skin organoids provide a physiologically relevant platform for modeling immune-stromal communication. *In vivo* models, including imiquimod-induced and IL-23-driven mouse models, together with lineage-tracing approaches, are essential for determining whether circulating immune cells differentiate into long-lived tissue-resident populations or whether inflammatory fibroblast subsets represent stable disease-associated states.

**Table 5:** Consensus annotation and validation markers of major cellular states in psoriasis.

Cell category	Cell state/subcluster	Consensus annotation marker	Dominant subtype	Recommended validation markers
Dendritic cells (7, 8, 14, 15, 28, 40, 41, 60)	pDC	CLEC4C, IL3RA, GZMB, TCF4	GP	CD123, CD303
	cDC1	CLEC9A, XCR1, BATF3, IRF8	PV	CLEC9A
	cDC2	CD1C, FCER1A, CLEC10A, HLA-DRA	PV	CD1c
	DC3	CD163, FCGR3A, S100A8, S100A9, IL1B	PV, PsA	CD163
	LAMP3^+^ mature DC	LAMP3, CCR7, CCL19	PV, PsA	LAMP3
Neutrophils (43–49)	Homeostatic neutrophil	FCGR3B, CXCR2, CSF3R, FPR1	GPP	MPO
	Inflammatory neutrophil	IL1B, CXCL8, S100A8, S100A9	GPP, PPP	MPO, S100A8
	NETosis neutrophil	MPO, ELANE, PADI4, CTSG	GPP	CitH3, PADI4
Monocytes/Macrophages (14, 15, 40, 55)	Classical monocyte	LYZ, FCN1, CTSS, S100A8	PV	CD14
	Inflammatory monocyte	CCR2, IL1B, TNF	PV, EP	CCR2
	Resident macrophage	C1QC, APOE, SELENOP, FOLR2	PV	CD68
	SPP1^+^ macrophage	SPP1, TREM2, APOE, GPNMB	PsA	SPP1
Mast cells (5, 6, 10, 26, 29–39, 42–51, 55, 57–62, 72, 73, 80)	Activated mast cell	KIT, TPSAB1, CPA3	PV	CD117
NK cells (63, 64)	Cytotoxic NK	NKG7, GNLY, KLRD1	PV	NKp46
ILCs (65)	ILC3	RORC, IL7R, KIT	PPP, GPP	CD127
T cells (52, 66–68)	Th17	CD4, RORC, CCR6, IL17A, IL17F	PV, GP	IL-17A
	Pathogenic Th17	IL23R, CSF2, IFNG	PV	GM-CSF
	Tc17	CD8A, IL17A, CXCR6, CCL20	PV, PsA	CD8, IL-17A
	Th1	TBX21, IFNG, CXCR3	PV, EP	T-bet
	Th22	IL22, AHR, CCR10	PV	IL-22
	Treg	FOXP3, IL2RA, CTLA4, TIGIT	PV	FOXP3
	Dysfunctional Treg	FOXP3, IL17A, IFNG	PV	FOXP3/IL17A
	TRM	CD69, ITGAE(CD103), CXCR6, ZNF683	All	CD69, CD103
Keratinocytes (14, 15, 28, 40)	Basal KC	KRT5, KRT14, COL17A1	All	KRT14
	Spinous KC	KRT1, KRT10	All	KRT10
	Proliferative KC	KRT6, KRT16, KRT17	All	KRT17
	Inflammatory KC	IL36G, S100A8, S100A9	GPP, PV	IL-36γ
	IFN-response KC	IFIT1, IFIT3, ISG15	GP	ISG15
Fibroblasts (14, 15, 40)	Papillary fibroblast	COL6A5, COL18A1, APCDD1	PV	COL6A5
	Reticular fibroblast	DPP4, COL1A1, MFAP5	PV	DPP4
	Inflammatory fibroblast	SFRP2, CCL13, CCL19, CXCL12, CTSS	PV	SFRP2
	Activated fibroblast	POSTN, COL11A1, TNC	PsA	POSTN

This table lists commonly used transcriptomic markers and recommended orthogonal validation markers for immune, epidermal, and stromal states, together with the phenotypes in which they have been reported.

Recent IMQ-based work has further demonstrated sex-dependent differences in psoriasis-like disease severity, with male BALB/c mice exhibiting stronger inflammatory and proliferative responses than females, underscoring the importance of incorporating sex as a biological variable in preclinical psoriasis modeling (81). IMQ-induced models have also been used to evaluate candidate combination interventions, including nutraceutical strategies, illustrating their utility for preclinical therapeutic screening while emphasizing that findings from murine models require subsequent validation in human tissue (82). Furthermore, multiplex immunofluorescence, imaging mass cytometry, spatial proteomics, and CITE-seq can validate the spatial organization and protein-level activity of candidate pathogenic cell populations identified by transcriptomic analyses. Integrating these complementary approaches will be critical for distinguishing computational predictions from biologically verified mechanisms and for translating single-cell discoveries into precision therapeutic strategies.

## 8 Therapeutic response and immune remodeling

Single-cell sequencing and spatial transcriptomic technologies are redefining the mechanisms of action of psoriasis therapies (72). Longitudinal transcriptomic studies suggest that disease remission may involve remodeling of the broader tissue immune ecosystem in addition to reductions in inflammatory cytokine expression. Reported changes include shifts in cellular composition, attenuation of inflammatory cell states, and computationally inferred alterations in intercellular communication. However, the relative contribution of each process to clinical remission remains uncertain.

### 8.1 Distinct immune remodeling patterns of different biologics

TNF inhibitors are thought to primarily act on the myeloid immune network. Single-cell studies have shown that after treatment, the activities of CCR2^+^ monocytes, SPP1^+^ macrophages, and LAMP3^+^ dendritic cells are markedly reduced, which is associated with decreased production of IL-23 and IL-1β; concurrently, the expression of inflammatory genes such as CXCL8, CCL20, and S100A8/A9 in keratinocytes is downregulated. Spatial transcriptomic observations suggest that transcriptional changes within dermal immune compartments may precede the contraction of epidermal inflammatory regions after TNF inhibition. This temporal association is consistent with, but does not establish, an upstream regulatory role for myeloid populations.

IL-17 inhibitors, by contrast, mainly act at the effector stage of the disease. After treatment, keratinocyte inflammatory programs are rapidly turned off, with expression of molecules such as CXCL1, CXCL8, CCL20, IL36G, and S100A7/A8/A9 declining markedly, and epidermal proliferation progressively returning toward homeostasis. However, TRM cells and Tc17 cells are not completely eliminated; some patients appear to retain a residual inflammatory niche, potentially in an IL-17 independent state, which may help explain the rapid relapse frequently observed after treatment discontinuation (14, 35, 38).

IL-23 blockade has provided an opportunity to examine the temporal process of disease resolution. In a limited number of longitudinal single-cell studies, early transcriptional changes were observed in inflammatory fibroblasts and myeloid cells, followed by attenuation of keratinocyte inflammatory programs and a more gradual reduction in Tc17- and TRM-associated activity. WNT5A^+^/IL24^+^ inflammatory fibroblasts showed early transcriptional regression, accompanied by reduced activity of LAMP3^+^ dendritic cells and CCR2^+^ macrophages. Predicted communication axes, including CXCL12-CXCR4 and CCL19-CCR7, were also attenuated during treatment (15).

These observations do not demonstrate that fibroblasts are direct targets of IL-23 inhibition or upstream regulators of therapeutic response. The early stromal changes may instead reflect rapid secondary responses to reduced downstream cytokine signaling. Moreover, the temporal sequence has been reported in only a limited number of cohorts and may vary according to treatment, sampling time, and analytical method. Larger longitudinal studies and functional experiments are therefore needed to determine whether tissue-microenvironment remodeling precedes adaptive immune deactivation or simply occurs in parallel with broader inflammatory network suppression.

**Figure 4:**
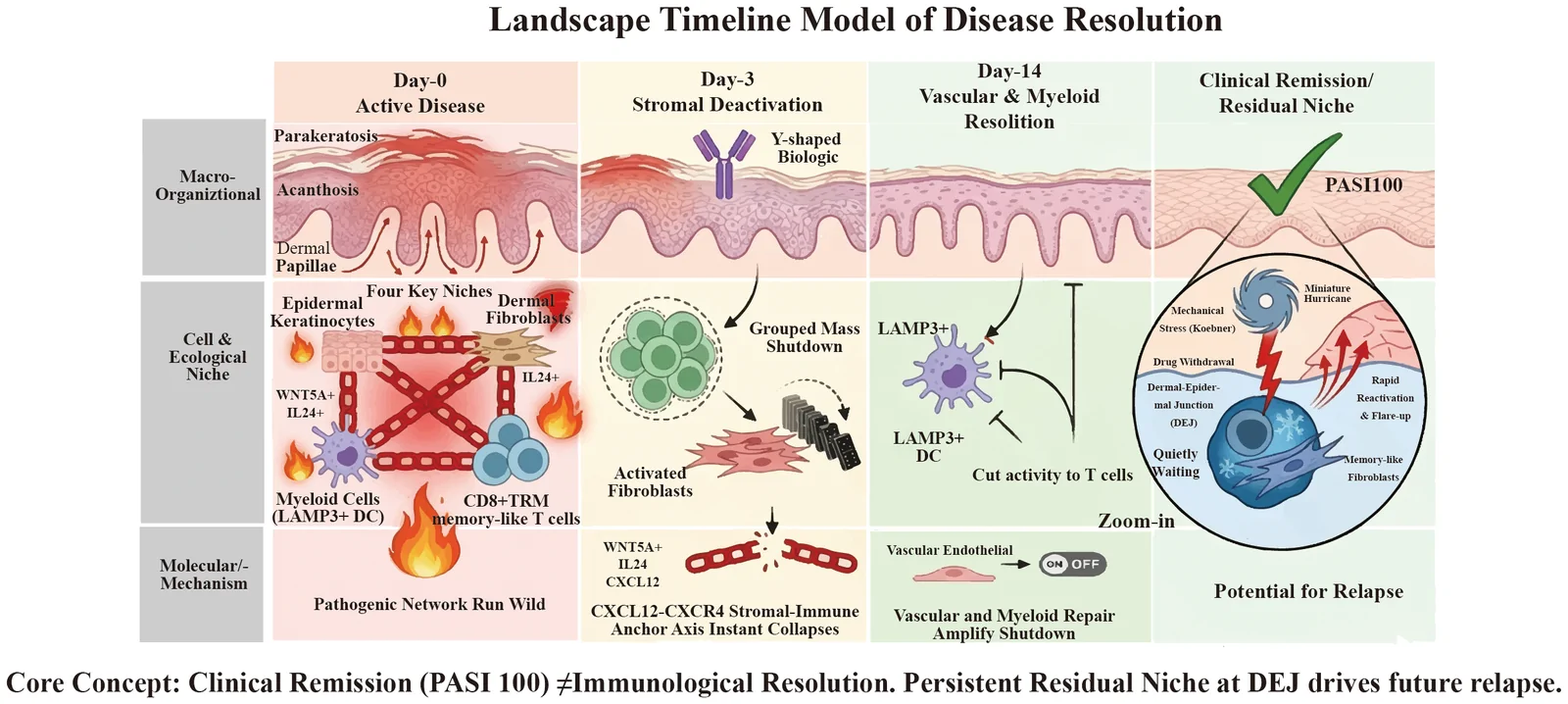
A proposed temporal model of therapeutic remodeling and residual inflammatory memory in psoriasis. Available longitudinal single-cell and spatial transcriptomic studies suggest a potential sequence of tissue remodeling during biologic therapy, including IL-23 blockade. The temporal stages illustrated here are derived from limited cohorts and should not be interpreted as a universal response pattern across all treatments or patients. At Day 0, active lesions are characterized by parakeratosis and acanthosis, together with extensive communication among epidermal, immune, stromal, and neurovascular niches. By approximately Day 3, early transcriptional changes may emerge in inflammatory fibroblasts, including WNT5A^+^/IL24^+^ subsets, and in selected myeloid populations. These stromal and innate immune changes may occur before more pronounced attenuation of adaptive immune programs. By approximately Day 14, vascular-remodeling and myeloid inflammatory signatures may be further reduced, accompanied by decreased activity or abundance of LAMP3^+^ dendritic cells and partial restoration of dermal-epidermal tissue organization. Even after complete clinical clearance, including PASI 100, residual molecular abnormalities may remain at previously affected sites. Tissue-resident memory T cells and fibroblasts with memory-associated transcriptional features have been proposed as components of this residual niche. Following treatment withdrawal or renewed exposure to local triggers, such as mechanical stress or infection, these residual cell states may contribute to rapid local inflammatory reactivation and lesion recurrence. However, their direct causal role in relapse requires confirmation through larger longitudinal cohorts and functional validation.

For GPP, IL-36 blockade may reduce neutrophil activation and NET-associated inflammatory amplification (47, 48). By disrupting the IL-36-neutrophil-IL-36 positive-feedback loop, it is thought to rapidly inhibit NETosis and reduce the expression of CXCL1, CXCL8, and IL1B, enabling rapid deconstruction of pustule-associated niches and restoration of tissue homeostasis (**Figure 4**).

Based on these findings, future efficacy evaluation may need to extend beyond the traditional PASI score and incorporate a new three-dimensional framework: remodeling of cellular composition, restoration of cell states, and deconstruction of spatial networks. Combined assessment of cellular composition, cell states, and spatial networks may provide a more stringent framework for defining deep remission (15, 40, 53).

## 9 Discussion

Single-cell RNA sequencing and spatial transcriptomics have shifted the conceptual framework of psoriasis from a disease explained predominantly by individual cytokine pathways toward a spatially organized and dynamically remodeled tissue immune ecosystem. The IL-23/IL-17 axis remains central to psoriasis pathogenesis, but current evidence indicates that its biological effects are mediated through coordinated interactions among immune cells, keratinocytes, fibroblasts, endothelial cells, and neurovascular components. This ecosystem-based framework helps explain why patients with similar clinical phenotypes or PASI scores may differ substantially in disease progression, treatment response, and recurrence.

A major contribution of single-cell technologies has been the recognition that cellular identity alone is insufficient to explain disease heterogeneity. Conventional classifications such as T cells, dendritic cells, macrophages, fibroblasts, and keratinocytes encompass multiple transcriptional and functional states. Pathogenic Tc17 and Th17 populations, long-lived tissue-resident memory T cells, LAMP3^+^ dendritic cells, SPP1^+^ macrophages, inflammatory fibroblasts, and IL36G-high keratinocytes may contribute differently across disease stages and clinical phenotypes. Nevertheless, many of these populations are currently defined primarily by transcriptional signatures. Whether they represent stable cell lineages, transient activation states, or continuous differentiation spectra remains incompletely resolved. This uncertainty is particularly relevant to T-cell biology. Th17, Tc17, Th1-like, and TRM populations frequently share overlapping inflammatory programs, and their developmental relationships cannot be determined reliably from cross-sectional transcriptomic data alone. Pseudo time and trajectory analyses may suggest potential transitions, but they do not establish direct lineage continuity. The proposed transition of Tc17 cells into long-lived TRM populations therefore remains a biologically plausible model rather than a fully demonstrated mechanism. Longitudinal sampling, T-cell receptor clonotype analysis, chromatin-accessibility profiling, and lineage-tracing approaches will be required to determine how pathogenic T-cell states are generated, maintained, and reactivated during recurrence.

Fibroblasts have similarly undergone an important conceptual reclassification. Single-cell studies indicate that fibroblasts are not merely structural cells but may participate in chemokine production, matrix remodeling, immune-cell recruitment, and spatial organization of inflammatory niches. However, fibroblast-subset nomenclature varies substantially among studies. SFRP2-, WNT5A-, IL24-, CXCL12-, and CCL19-associated populations are not always defined consistently, partly because of differences in sampling depth, anatomical location, clustering resolution, and marker selection. It therefore remains uncertain whether inflammatory fibroblasts constitute stable disease-specific populations or reversible states induced by the local cytokine environment. Functional perturbation and longitudinal tissue analysis will be necessary before fibroblast-targeted therapeutic strategies can be considered clinically realistic.

Spatial transcriptomics adds an essential anatomical dimension to these cellular findings. Psoriatic inflammation appears to be organized into partially distinct epidermal, dermal immune, stromal, vascular, and neuroimmune compartments. The dermal-epidermal junction may contain persistent TRM-associated inflammatory programs, whereas deeper dermal and perivascular regions may be enriched in mature dendritic cells, myeloid populations, and activated fibroblasts. These observations support the concept that psoriasis is maintained by spatially restricted pathological niches rather than by uniformly distributed inflammatory cells. However, spatial colocalization does not itself demonstrate direct cellular communication. Many proposed ligand-receptor interactions, including CXCL12-CXCR4, CCL19-CCR7, and IL24-related signaling, remain based on computational inference and should be interpreted as hypothesis-generating rather than definitive mechanistic evidence.

Differences among psoriasis phenotypes further demonstrate that the disease cannot be reduced to a single universal immune architecture. Plaque psoriasis is associated with a chronic adaptive immune-stromal maintenance network involving Th17/Tc17 cells, TRM cells, dendritic cells, fibroblasts, and keratinocytes. Guttate psoriasis may represent an acute, infection-associated inflammatory state, but whether it is a distinct phenotype or a transitional stage toward plaque psoriasis remains controversial. Generalized pustular psoriasis is more strongly linked to IL-36-mediated autoinflammation and neutrophilic amplification, whereas palmoplantar pustulosis appears to involve a site-specific palmoplantar microenvironment. Erythrodermic psoriasis shows broad systemic immune activation, but current studies cannot determine whether the observed molecular abnormalities are primary drivers or secondary consequences of severe inflammation. In psoriatic arthritis, shared T-cell clonotypes and inflammatory pathways across skin and synovium support a skin-joint immune axis, although direct cellular migration and causal relationships remain unproven.

Interpretation of these subtype-specific findings is limited by the uneven distribution of available evidence. Most single-cell and spatial datasets have been generated from plaque psoriasis, whereas guttate, pustular, erythrodermic, palmoplantar, and arthritic phenotypes remain underrepresented. Cohort sizes are generally small, and differences in disease duration, anatomical sampling, treatment exposure, tissue processing, sequencing platform, and computational pipeline complicate direct comparison. Consequently, some apparent subtype-specific cell states may reflect technical variation, sampling bias, or differences in disease stage rather than fundamental biological distinctions. Larger multicenter studies using harmonized clinical definitions and analytical frameworks are needed to establish a reproducible molecular taxonomy of psoriatic disease.

Longitudinal treatment studies provide an initial framework for understanding how the psoriatic ecosystem resolves. Current evidence suggests that successful treatment may involve coordinated changes in myeloid cells, fibroblasts, keratinocytes, and pathogenic T-cell populations rather than simple suppression of a single cytokine. Nevertheless, the temporal sequence of these changes varies among studies and should not automatically be interpreted as a causal hierarchy. Early transcriptional changes in fibroblasts or myeloid cells after IL-23 blockade, for example, do not establish that these cells are direct therapeutic targets or upstream regulators of clinical response. Similarly, reductions in CellChat derived communication scores after treatment may reflect changes in cellular abundance or transcriptional activity rather than direct disruption of functional signaling networks.

The persistence of molecular abnormalities after visible lesion clearance raises the possibility that clinical remission and biological remission are not equivalent. TRM cells and residual stromal or keratinocyte-associated inflammatory programs may remain in clinically resolved skin and could contribute to localized relapse. However, the causal role of these residual niches has not been conclusively demonstrated. It remains unclear whether they actively initiate recurrence, merely mark previously inflamed tissue, or require additional systemic and environmental triggers to regain pathogenic activity. Prospective longitudinal studies sampling lesions before treatment, during remission, and at recurrence will be critical for determining which residual molecular features genuinely predict relapse. These findings also have important translational implications, but their current clinical maturity should not be overstated. Single-cell and spatial omics are unlikely to replace routine clinical diagnosis or conventional disease assessment in the near term. Their more immediate value lies in identifying reproducible cell-state signatures, spatial biomarkers, and simplified tissue or circulating markers that may improve patient stratification, treatment-response prediction, and relapse-risk assessment. Translation will require conversion of complex discovery-level signatures into practical assays, such as targeted transcript panels, multiplex immunostaining, minimally invasive skin sampling, or validated circulating biomarkers.

Overall, the spatial immune-ecosystem model provides a useful framework for integrating immune activation, tissue remodeling, disease heterogeneity, therapeutic response, and recurrence in psoriasis. However, many components of this model remain provisional because they are derived from cross-sectional transcriptomic associations, computational predictions, and relatively small cohorts. The next stage of the field should therefore move beyond increasingly detailed descriptive atlases toward longitudinal, multimodal, and experimentally validated models that distinguish causal mechanisms from secondary inflammatory adaptations. Such work will be necessary to determine which cell states and spatial niches are biologically reproducible, therapeutically targetable, and clinically actionable.

## 10 From spatial immune ecosystems to clinical precision medicine

Single-cell and spatial omics have the potential to connect the biological heterogeneity of psoriasis with clinically meaningful classification, monitoring, and treatment strategies. However, most currently proposed applications remain at the discovery or exploratory stage. Their clinical value will depend on whether molecular signatures can be reproduced across independent cohorts, measured using practical assays, and shown to improve clinical decision-making beyond established clinical and laboratory parameters (14, 15, 28, 40, 53, 77).

### 10.1 Molecular diagnosis and disease classification

Psoriasis is usually diagnosed clinically and, when necessary, supported by histopathological examination. Therefore, single-cell or spatial transcriptomic testing is unlikely to replace routine clinical diagnosis. Its more realistic diagnostic value may lie in resolving atypical or overlapping inflammatory skin diseases and refining the molecular classification of clinically heterogeneous phenotypes (83). Cell-state and spatial signatures may help distinguish plaque psoriasis from pustular phenotypes, erythrodermic psoriasis, psoriatic arthritis, and other inflammatory dermatoses with partially overlapping clinical or histopathological features. For example, plaque psoriasis is generally associated with coordinated Th17/Tc17, tissue-resident memory T-cell, dendritic-cell, fibroblast, and keratinocyte programs, whereas generalized pustular psoriasis is characterized by a more prominent IL-36-centered neutrophilic inflammatory network. Palmoplantar pustulosis may exhibit a site-specific inflammatory niche involving sweat duct epithelium, keratinocytes, neutrophils, and plastic T-cell states. Nevertheless, these molecular distinctions remain insufficiently standardized for diagnostic use. Research-derived signatures must therefore be converted into simplified, reproducible, and clinically validated assays before they can support routine disease classification (14, 15, 28–30, 32, 33, 40, 41, 51, 54, 57, 59, 77).

### 10.2 Patient stratification

Single-cell profiling may enable patients to be stratified according to dominant pathogenic cellular programs rather than clinical morphology alone (14, 15, 28, 33, 40, 41, 57, 59). Lesions with similar clinical severity may differ substantially in their relative enrichment of Th17/Tc17-associated inflammation, IL-36 neutrophil activity, interferon-responsive programs, inflammatory myeloid cells, or activated fibroblast populations. Spatial transcriptomics may further distinguish epidermal-dominant, dermal immune, stromal, neurovascular, and residual memory niches. Such molecular stratification may help explain why patients with comparable Psoriasis Area and Severity Index scores exhibit different patterns of disease progression, comorbidity, therapeutic response, and recurrence. For example, patients with a predominantly IL-36-neutrophil inflammatory program may differ biologically from those with a stable TRM-fibroblast inflammatory niche, even when both present with extensive cutaneous inflammation. Similarly, the detection of shared T-cell states or clonotypes across skin and synovium may help define patients at greater risk of joint involvement. However, current cell-state classifications remain inconsistent across sequencing platforms, tissue-processing protocols, and computational pipelines. Prospective multicenter studies will therefore be required to determine whether molecularly defined subgroups are reproducible and clinically actionable.

### 10.3 Biomarker discovery

Single-cell and spatial analyses provide a framework for identifying biomarkers that may be obscured in bulk tissue measurements (14, 15, 28, 40, 41, 53). Candidate biomarkers include the abundance, activation state, or spatial organization of Tc17 cells, TRM cells, LAMP3^+^ dendritic cells, SPP1^+^ macrophages, inflammatory fibroblasts, and IL36G-high keratinocytes. Composite signatures incorporating multiple cell states and signaling pathways may prove more informative than individual cytokines because they could capture the broader organization of the inflammatory ecosystem. Spatially restricted biomarkers may also provide information beyond total gene expression. For example, the localization of TRM cells near the dermal-epidermal junction, the enrichment of inflammatory fibroblasts in stromal niches, or the colocalization of dendritic cells and pathogenic T-cell populations may be associated with disease persistence or recurrence. Nevertheless, tissue-based biomarkers are invasive and may vary according to lesion site, disease stage, and previous treatment. Clinical translation will therefore require simplified immunohistochemical or multiplex imaging panels, targeted transcript assays, minimally invasive skin-sampling approaches, or circulating surrogate markers that reliably reflect tissue-level pathogenic states.

### 10.4 Prediction of therapeutic response

Longitudinal single-cell studies suggest that targeted therapies may induce distinct temporal patterns of immune and stromal remodeling. Baseline cellular and molecular profiles could therefore potentially identify patients who are more likely to respond to IL-17, IL-23, TNF, or IL-36 pathway inhibition. For example, strong IL-36- and neutrophil-associated programs may support the biological relevance of IL-36-targeted therapy in pustular disease, whereas persistent IL-23/IL-17, TRM, and fibroblast-associated networks may influence treatment responses in chronic plaque psoriasis. Early transcriptional changes in inflammatory fibroblasts, myeloid populations, keratinocytes, or pathogenic T-cell states may also serve as pharmacodynamic indicators before complete clinical improvement becomes apparent. Computational analyses have further suggested that successful treatment may be accompanied by reduced ligand-receptor communication and partial deconstruction of inflammatory niches. However, these inferred communication changes do not directly demonstrate functional network disruption. In addition, most proposed response signatures are derived from relatively small cohorts and should not yet be regarded as validated predictive biomarkers. Their clinical utility must be tested in prospective studies comparing molecularly guided treatment selection with current standard of care decision-making (15, 40, 47, 48).

### 10.5 Disease monitoring and relapse prediction

Clinical scores such as PASI primarily measure visible disease severity and may not fully capture residual molecular inflammation (40). Single-cell and spatial studies indicate that clinically resolved lesions can retain TRM cells and low-level inflammatory or stromal programs. These residual cellular niches may contribute to localized recurrence, although their causal importance has not yet been definitively established. Molecular monitoring could therefore complement conventional clinical assessment by evaluating the persistence of pathogenic cell states, restoration of epidermal and stromal homeostasis, and attenuation of spatial inflammatory networks. Potential monitoring markers may include residual TRM-associated signatures, persistent inflammatory fibroblast states, incomplete normalization of keratinocyte programs, or continued enrichment of myeloid and dendritic-cell populations. Such measures could help distinguish superficial clinical improvement from deeper molecular remission.

Repeated full-thickness skin biopsies, however, are impractical for routine follow-up. Future monitoring strategies will therefore need to use minimally invasive approaches, such as tape stripping, microneedle-based sampling, targeted skin swabs, suction-blister fluid, or validated circulating biomarkers. Longitudinal studies are also required to determine whether residual molecular signatures can reliably predict relapse after treatment withdrawal or treatment-interval extension.

### 10.6 Toward precision medicine

A clinically useful precision-medicine framework should integrate conventional clinical variables with molecular information rather than replace established assessment systems. Such a model could combine clinical phenotype, disease duration, comorbidities, previous treatment exposure, cellular composition, pathogenic cell-state activity, spatial niche characteristics, immune-receptor clonality, and longitudinal molecular changes. This integrated framework could potentially support molecular patient stratification, treatment selection, early response assessment, recurrence-risk estimation, and identification of patients who require prolonged or intensified therapy. Future therapeutic endpoints may also extend beyond visible lesion clearance to include normalization of pathogenic cell states, reduction of residual TRM-associated signatures, restoration of stromal and epidermal homeostasis, and attenuation of persistent pathological niches (16–20, 77, 84).

Substantial barriers remain before these approaches can be incorporated into routine psoriasis management. These include the cost and complexity of omics technologies, tissue-sampling requirements, platform-dependent variability, small cohort sizes, underrepresentation of rare clinical phenotypes, and the absence of standardized analytical, regulatory, and reporting frameworks. Most current molecular signatures are also based on research-grade assays that are not readily transferable to clinical laboratories. Multicenter prospective cohorts, harmonized protocols, simplified biomarker panels, independent validation, and demonstration of added clinical value beyond established measures such as PASI will therefore be essential.

Overall, single-cell and spatial omics provide a conceptual and technical foundation for redefining psoriasis as a heterogeneous spatial immune ecosystem. Their most immediate clinical contribution is likely to arise not from routine whole-transcriptome testing, but from the development of focused biomarkers and practical assays derived from reproducible cellular and spatial signatures. Such approaches may ultimately enable more accurate disease stratification, individualized therapeutic selection, molecular monitoring, and durable disease control.

## 11 Future perspectives: ai-enabled spatial multi-omics and longitudinal disease mapping

The next stage of psoriasis research will require a transition from isolated, cross-sectional transcriptomic observations toward integrated and longitudinal models capable of linking molecular regulation, tissue architecture, clinical phenotype, and therapeutic outcome. Artificial intelligence, machine learning, spatial multi-omics, and digital pathology may provide important tools for achieving this transition. However, their value will depend on the quality, diversity, interpretability, and clinical validation of the underlying data.

### 11.1 Artificial intelligence-assisted transcriptomic analysis

The increasing scale and complexity of single-cell and spatial transcriptomic datasets have created analytical challenges that cannot be fully addressed using conventional statistical approaches alone. Artificial intelligence and machine-learning models may assist with cell-state annotation, dimensionality reduction, multimodal data integration, spatial-domain recognition, trajectory reconstruction, and identification of complex molecular patterns associated with disease phenotype or therapeutic response.

Foundation models and other representation-learning approaches may eventually enable knowledge transfer across datasets, platforms, tissues, and inflammatory diseases. Such models could help identify rare pathogenic cell states, harmonize heterogeneous annotations, and reveal shared or subtype-specific regulatory programs. Nevertheless, AI-generated cellular classifications and biological hypotheses remain dependent on the quality and representativeness of the training data. Models trained predominantly on plaque psoriasis or selected patient populations may perform poorly in rare phenotypes, different anatomical sites, or underrepresented demographic groups. Therefore, AI-assisted analysis should complement rather than replace biological interpretation and independent experimental validation (20, 85, 86).

### 11.2 Machine learning for biomarker discovery and treatment prediction

Machine-learning approaches may facilitate the identification of composite biomarkers by integrating transcriptomic signatures with cellular composition, spatial organization, clinical variables, and treatment outcomes. Compared with individual cytokines or genes, multidimensional signatures may better represent the complexity of psoriatic inflammation and may improve patient stratification, prediction of therapeutic response, and estimation of relapse risk.

Potential predictors may include the abundance or activation state of Tc17 and TRM cells, inflammatory fibroblast and myeloid-cell programs, IL-36-associated neutrophilic signatures, spatial immune-neighborhood characteristics, and early molecular changes after treatment. However, biomarker models derived from small retrospective datasets are vulnerable to overfitting, data leakage, batch effects, and optimistic estimates of predictive performance. Future models should therefore be developed using patient-level training and validation, tested in external multicenter cohorts, compared against established clinical predictors, and evaluated prospectively before clinical implementation.

### 11.3 Spatial multi-omics integration

Spatial transcriptomics provides information on gene expression and tissue organization but does not fully capture chromatin regulation, protein abundance, metabolic activity, or functional signaling. Spatial multi-omics approaches that combine transcriptomic, epigenomic, proteomic, metabolomic, histological, and immune-receptor information may enable more complete characterization of psoriatic pathological niches.

For example, integrating spatial transcriptomics with chromatin-accessibility data may help identify the regulatory programs that establish persistent TRM or inflammatory fibroblast states. Spatial proteomics may determine whether predicted ligand-receptor pairs are present at the protein level, whereas metabolomic profiling may reveal local metabolic conditions that support pathogenic immune-cell or keratinocyte states. T-cell receptor sequencing can further link spatially localized T-cell populations with clonal expansion and tissue persistence.

Despite this potential, spatial multi-omics integration remains technically and computationally challenging. Different modalities vary in spatial resolution, sensitivity, dynamic range, tissue compatibility, and measurement scale. Some measurements are obtained from the same tissue section, whereas others require adjacent sections or computational alignment. Consequently, apparent multimodal associations may reflect registration errors, sampling differences, or algorithmic assumptions. Standardized benchmarking and orthogonal validation will be necessary to determine whether integrated spatial models reflect reproducible biological relationships (84).

### 11.4 Digital pathology and multimodal tissue analysis

Digital pathology provides an opportunity to connect conventional histopathology with molecular and spatial data. Whole-slide imaging and computational image analysis may quantify epidermal thickness, parakeratosis, vascular remodeling, inflammatory-cell density, and the distribution of pathological tissue compartments. These image-derived features could be integrated with spatial transcriptomic or proteomic measurements to identify morphological correlates of molecular cell states and inflammatory niches.

Such multimodal analysis may ultimately support the development of clinically accessible surrogate markers. Rather than performing whole-transcriptome profiling in every patient, validated image-based models could potentially recognize histological patterns associated with particular inflammatory programs, treatment responses, or residual disease states. However, digital pathology models require carefully annotated and standardized tissue collections. Variation in biopsy orientation, fixation, staining, scanning equipment, tissue quality, and pathological interpretation may substantially affect model performance. Transparent reporting, external validation, and human oversight will remain essential.

### 11.5 Longitudinal tissue mapping

Most current psoriasis single-cell and spatial studies provide cross-sectional snapshots of established lesions. These designs cannot reliably determine the temporal order of immune activation, stromal remodeling, treatment response, molecular remission, and recurrence. Longitudinal tissue mapping will therefore be essential for reconstructing the natural history of the psoriatic immune ecosystem (15, 20, 53, 77).

Ideally, future studies should collect matched lesional, non-lesional, resolving, and recurrent tissue from the same patients before treatment and at defined follow-up time points. These samples should be integrated with clinical severity, treatment exposure, anatomical site, comorbidities, and long-term outcomes. Such designs could determine whether fibroblast and myeloid-cell changes consistently precede adaptive immune remodeling, whether residual TRM-associated states predict local relapse, and whether clinically resolved skin returns to a genuinely homeostatic state (15).

Repeated conventional biopsies may be difficult to implement because of patient burden and tissue-site variability. Longitudinal research may therefore require combinations of limited tissue biopsy, tape stripping, suction-blister sampling, microneedle-based collection, blood biomarkers, and non-invasive imaging. Analytical approaches must also distinguish true temporal change from variation caused by repeated sampling, anatomical heterogeneity, treatment adherence, and technical batch effects (16, 18, 20).

### 11.6 Challenges in clinical translation

Several barriers must be addressed before AI-assisted and spatial multi-omics approaches can influence routine psoriasis care. First, the cost, infrastructure, computational requirements, and tissue-processing demand of these technologies currently limit their use in large clinical cohorts. Second, differences in platforms and analytical pipelines hinder reproducibility and cross-study comparison. Third, many available datasets contain limited clinical annotation and disproportionately represent plaque psoriasis, reducing the generalizability of resulting models.

Data governance is another important consideration. Multimodal models may combine genomic, histological, imaging, and longitudinal clinical information, creating challenges related to privacy, data sharing, algorithmic bias, and regulatory oversight. Clinically deployed models should provide interpretable results, demonstrate consistent performance across populations and institutions, and undergo prospective evaluation against meaningful clinical outcomes. The most realistic route to clinical implementation may therefore involve translating complex discovery-level datasets into focused and standardized assays. These could include targeted gene panels, simplified multiplex immunostaining, digital histological scores, circulating biomarkers, or clinically interpretable composite models. Large multicenter cohorts, harmonized protocols, open analytical pipelines, independent validation, and health-economic evaluation will be required to determine whether these approaches improve patient outcomes beyond established clinical assessment and treatment-selection strategies.

Overall, artificial intelligence and spatial multi-omics offer a potential framework for transforming psoriasis research from descriptive cellular mapping toward predictive and clinically actionable disease modeling. Their successful application will require close integration among computational scientists, immunologists, dermatopathologists, clinical dermatologists, and patients. Most importantly, computational performance must ultimately be judged by whether it improves biological understanding, therapeutic decision-making, and durable disease control.

## 12 Conclusion

Single-cell and spatial omics have shifted the conceptual framework of psoriasis from a disease dominated by isolated cytokine pathways toward a spatially organized and dynamically remodeled tissue immune ecosystem. Within this framework, pathogenic inflammation emerges from coordinated changes in immune cells, keratinocytes, fibroblasts, endothelial cells, and neurovascular components. These technologies have also revealed substantial heterogeneity across clinical phenotypes and have highlighted cell states, tissue neighborhoods, and residual inflammatory niches as potentially important determinants of disease persistence, treatment response, and recurrence.

Several fundamental biological questions remain unresolved. It is unclear which cell populations initiate the psoriatic ecosystem, how Th17, Tc17, and TRM states are developmentally related, whether inflammatory fibroblasts are stable lineages or reversible states, and whether residual immune–stromal niches directly cause local recurrence. Models derived predominantly from plaque psoriasis also cannot yet be generalized to guttate, pustular, erythrodermic, palmoplantar, or arthritic phenotypes.

The immediate priority is to test these questions in longitudinal, multimodal, and experimentally tractable designs. Matched sampling of lesional, non-lesional, resolving, and recurrent skin, integrated with chromatin accessibility, surface-protein profiling, spatial proteomics, immune-receptor sequencing, and digital pathology, could reconstruct pathogenic trajectories. Candidate mechanisms should then be examined in co-culture, organotypic skin, lineage-tracing, and perturbation models, using standardized processing and independent validation across rare phenotypes.

Important barriers also remain between molecular discovery and clinical implementation. Whole-transcriptome and spatial multi-omics assays are currently limited by cost, technical complexity, tissue requirements, and the need for specialized computational infrastructure. Repeated biopsies are unlikely to be practical for routine monitoring, and most proposed molecular signatures have not demonstrated predictive value beyond established clinical measures. Translation will therefore depend on converting complex discovery-level signatures into focused and clinically feasible assays, such as targeted transcript panels, simplified multiplex imaging, minimally invasive skin sampling, circulating surrogate biomarkers, or validated digital pathology models. These tools will require prospective multicenter evaluation, standardized performance thresholds, regulatory oversight, and evidence that they improve treatment selection, disease monitoring, or relapse prediction.

Accordingly, the principal value of single-cell and spatial omics lies not only in generating increasingly detailed maps of psoriatic tissue, but in defining which cellular states and spatial relationships are reproducible, causal, measurable, and clinically actionable. Achieving this goal will require coordinated longitudinal, multi-omics, functional, and translational studies. Such efforts may ultimately enable psoriasis management to progress from phenotype-based disease control toward biologically informed patient stratification, molecular monitoring, and more durable remission.
